# Hsp70 Interacts with the TREM-1 Receptor Expressed on Monocytes and Thereby Stimulates Generation of Cytotoxic Lymphocytes Active against MHC-Negative Tumor Cells

**DOI:** 10.3390/ijms22136889

**Published:** 2021-06-26

**Authors:** Tatiana N. Sharapova, Elena A. Romanova, Olga K. Ivanova, Denis V. Yashin, Lidia P. Sashchenko

**Affiliations:** Laboratory of Molecular Immunogenetics of Cancer, Institute of Gene Biology RAS, Vavilova 34/5, 111394 Moscow, Russia; elrom4@rambler.ru (E.A.R.); olga.k.ivanova@gmail.com (O.K.I.); yashin_co@mail.ru (D.V.Y.); sashchenko@genebiology.ru (L.P.S.)

**Keywords:** monocytes, Hsp70, NK, cytotoxicity, cytokine

## Abstract

The search for and analysis of new ligands for innate immunity receptors are of special significance for understanding the regulatory mechanisms of immune response. Here we show that the major heat shock protein 70 (Hsp70) can bind to and activate TREM-1, the innate immunity receptor expressed on monocytes. The Hsp70–TREM-1 interaction activates expression of TNFα and IFNγ mRNAs in monocytes and stimulates IL-2 secretion by PBMCs. Moreover, incubation of PBMCs with Hsp70 leads to an appearance of cytotoxic lymphocyte subpopulations active against the MHC-negative tumor cells. In addition, both the CD4+ T-lymphocytes and CD14+ monocytes are necessary for the Hsp70 signal transduction and a consequent activation of the cytotoxic lymphocytes. We believe that data presented in this study will broaden the views on the involvement of Hsp70 in the antitumor immunity.

## 1. Introduction

The elucidation of regulatory mechanisms of immune response and identification of the new regulatory molecules are extremely important for gaining an insight into the molecular basis of immunity and for developing effective approaches to immunotherapy. Moreover, it is essential to search not only for the new regulatory proteins but also for their specific receptors located on the surface of immune cells, through which they induce intracellular signal transduction and the consequent activation of these cells.

The initial stages of immune response involve the activation of innate immunity cells, and it is therefore important to study receptors involved in innate immunity. The best studied among them is the family of the Toll-like receptors (TLRs) [1]. The TLRs-induced signaling stimulates production of interferons and proinflammatory cytokines involved in the antibacterial defense [2]. A family of the Triggering Receptors Expressed on Myeloid cells (TREMs) was described in 2000 [3], they play an important role in the modulation of immune response. They are regarded as crucial regulators of various cellular functions, including the enhancement of inflammation [4,5]. Different representatives of the TREM family have opposite effects on cells (i.e., this family includes both the activating and the inhibitory receptors) [6].

The best known activating receptor is TREM-1, which is expressed on the surface of monocytes, neutrophils, granulocytes, dendritic cells, NK cells, and, at a low level, also in T cells and some B-cell subpopulations [7,8]. It was thought for some time that the main function of TREM-1 was to modify the immune defense against bacteria and fungi during development of infections. Subsequent studies have established that TREM-1 is involved in the regulation of the T-cell proliferation, activation of antigen-presenting cells (APCs), and the antiviral defense [5]. TREM-1 is a transmembrane protein that has an Ig-like ectodomain responsible for ligand binding [9,10], a short transmembrane domain, and a cytoplasmic tail necessary for signal transduction. Its interaction with the DAP12 adaptor molecule triggers sequential phosphorylation of the cascade of tyrosine protein kinases with a consequent activation of PIK3 phospholipase and transcription factors responsible for the expression of genes encoding proinflammatory cytokines [11,12]. Soluble TREM-1 (sTREM-1) has also been described [3,4,11]. It appears to be able to block the transduction of TREM-1-dependent signal, but its functions have not been elucidated in detail.

Although identification of ligands for any receptor is crucial for understanding the relationship between signaling from the receptor and pathogenesis of a disease, TREM-1 ligands have not been studied sufficiently. Three proteins are currently regarded as probable ligands for this receptor. One of them is a high-mobility group Box 1 protein (HMGB1), a nuclear protein that can interact with the nucleosomal histones and transcription factors, thereby participating in the of transcription. HMGB1 can initiate signaling through different receptors, including TLRs and RAGE. It has been shown that HMGB1 and TREM-1 can directly interact with each other [13]. Cell lysates from the necrotic human monocytes contained HMGB1 which can induce proinflammatory response. A specific TREM-1 inhibitor aborts this effect [14]. These observations provide some evidence that HMGB1 could be a ligand for TREM-1.

The innate immunity protein PGLYRP1 was also identified as a ligand for TREM-1. In experiments where the immunoprecipitation of cell lysates prepared from the peptidoglycan-activated neutrophils with TREM-1 tetramer and a subsequent mass spectrometry analysis have revealed that PGLYRP1 binds to TREM-1 [15]. The data obtained in our laboratory also confirm that PGLYRP1 may be regarded as a ligand for TREM-1. We have shown that the recombinant innate immunity protein Tag7 (a.k.a. PGLYRP1) induces TREM-1-dependent signaling, and that Tag7 interaction with human monocytes leads not only to the enhanced expression of genes coding for proinflammatory cytokines but also to the appearance of lymphocyte subpopulations, showing cytotoxic activity against MHC-negative tumor cells [16].

There are several lines of evidence suggesting that Hsp70 could also be regarded as a potential ligand for TREM-1. This protein functions mainly as a molecular chaperone responsible for the maintenance of protein homeostasis under normal and stress conditions. The ability of Hsp70 to regulate the immune response was also suggested. In particular, it has been found that lysates of necrotic human monocyte cells containing both HMGB1 and Hsp70 can induce proinflammatory signals that are blocked by TREM-1 and Hsp70 inhibitors. However, the direct binding of Hsp70 to TREM-1 has not been detected previously and the hypothesis suggesting functional interactions between these proteins is only supported by the fact that the TREM-1-induced signaling is blocked by the antibodies to Hsp70 [13,14].

The purpose of this study was to find out whether Hsp70 is actually a ligand for the innate immunity receptor TREM-1 and what is the role of Hsp70 in activation of cytotoxic lymphocytes and antitumor immunity.

## 2. Results

### 2.1. Hsp70 Binds to TREM-1

**Figure 1:**
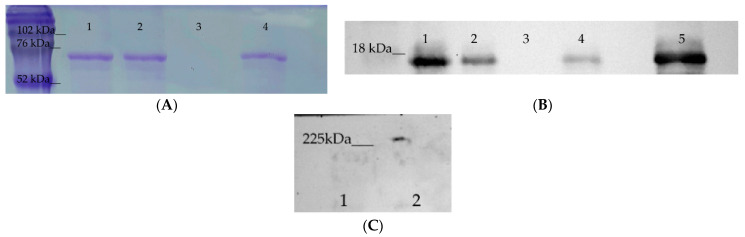
Hsp70 binding to TREM-1. (**A**) Hsp70 binding to sTREM-1 immobilized on a CNBr-Sepharose column (SDS-PAGE with Coomassie staining): (1) Control Hsp70, (2) unbound fraction of Hsp70 loaded to column, (3) washed fraction from column before elution, (4) Hsp70 eluted by TEA from the column. (**B**) TREM-1 binding with Hsp70 immobilized on a CNBr-Sepharose column (SDS-PAGE followed by western blotting with specific anti-TREM-1 antibodies. (1) Fraction washed with PBS, (2) fraction washed with PBS + 0.5 M NaCl, (3) fraction washed with PBS before elution, (4) TREM-1 eluted by TEA from the column, (5) control TREM-1. (**C**) Hsp70 binding to TREM-1 on the monocyte surface. Monocytes were incubated with Hsp70 in the presence of BS3, lysed, and purified using Dynabeads conjugated with anti-Hsp70 antibodies. This material was resolved by 10% PAGE followed by western blotting with anti-TREM-1 antibodies and visualized using the ECL plus detection Kit: (1) RPN 800E marker, (2) purified Hsp70–TREM-1 fraction. (Original blots are in Supplementary materials).

The ability of Hsp70 to interact with TREM-1 was analyzed using affinity chromatography. Two CNBr-activated Sepharose 4B columns were prepared, one with immobilized sTREM-1 and the other with immobilized Hsp70. A sample of Hsp70 was applied onto the first column, and the bound protein was eluted and resolved by the SDS-PAGE. The eluate was found to contain Hsp70 (Figure 1A), which indicated an interaction of this protein with the TREM-1 immobilized on the column. This interaction was confirmed by the results of chromatography of the soluble sTREM-1 (ectodomain) on the column with the immobilized Hsp70. The eluate from this column was resolved by SDS-PAGE followed by western blotting with a specific anti-TREM-1 antibodies (Figure 1B). The BSA was used as a negative control to exclude unspecific binding with CNBr-Sepharose and it was not detected in the fractions with elution material (Supplement 1). We performed additional experiments in order to prove the specificity of the TREM-1 binding to Hsp70 (Supplement 2). As shown in (Supplement 2), we have detected sTREM-1 after incubation of Hsp70-sTREM-1 with the anti-Hsp70 couple beads.

We then tested for the possibility of Hsp70 binding to TREM-1 expressed on the monocyte cell membrane. Monocytes were incubated with Hsp70 in the presence of BS^3^ crosslinking reagent, lysed, and purified by magnetic separation using Dynabeads conjugated with anti-Hsp70 antibodies (abHsp70-Dynabeads). This material was resolved by 10% PAGE followed by western blotting with specific anti-TREM-1 antibodies. It can be seen that the fraction purified with abHsp70-Dynabeads contained a protein complex interacting with anti-TREM-1 antibodies, which indicated that it contained both Hsp70 and TREM-1 (Figure 1C). The molecular mass of this complex was approximately 200 kDa, exceeding that of the equimolar Hsp70–TREM-1 complex (70 + 26 kDa). A probable explanation is that Hsp70 binds to TREM-1 tetramer expressed on the cell surface (70 + 104 kDa) or that two Hsp70 molecules are bound with TREM-1 dimer in this complex (140 + 52 kDa) (Figure 1C).

Thus, Hsp70 can bind to TREM-1. To verify that Hsp70 is actually a ligand for TREM-1, we analyzed what effect this binding may have on TREM-1-dependent signaling during activation of the cytotoxic lymphocytes involved in the innate and adaptive immune responses.

### 2.2. Hsp70 Activates Cytotoxic Lymphocytes Reacting against MHC-Negative Tumor Cells

The next step was to find out whether the Hsp70–TREM-1 interaction leads to an induction of the cytotoxic lymphocyte subpopulations, as was observed in our previous study on the mechanism of action of the innate immunity protein Tag7, another TREM-1 ligand [16].

**Figure 2:**
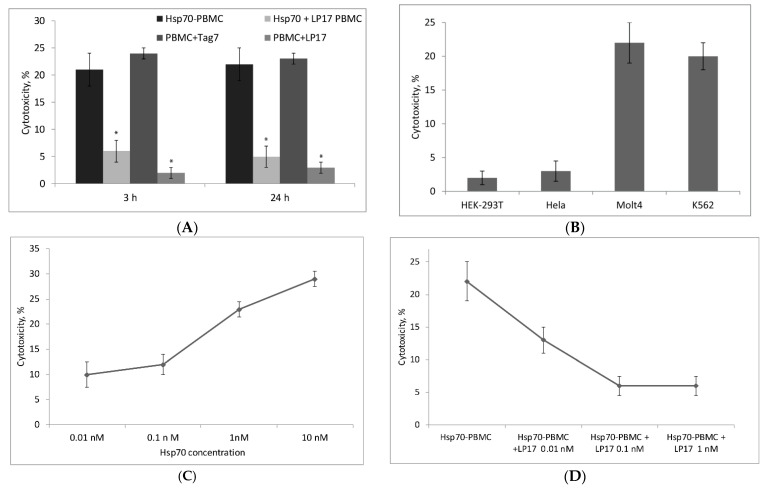
Cytotoxic activity of Hsp70-activated lymphocytes. (**A**) Cytotoxic activity against K562 cells of lymphocytes activated in the PBMC (peripheral blood mononuclear cells) pool by six-day treatment with Hsp70, LP17 + Hsp70, LP17 only, Tag7. (**B**) Cytotoxic activity of Hsp70-activated lymphocytes against four different cell lines (HEK-293T, HeLa, Molt4, and K562). (**C**) Dependence of cytotoxicity of activated lymphocytes on Hsp70 concentration during six-day treatment. (**D**) Inhibition of cytotoxicity of Hsp70-activated lymphocytes depending on the LP17 concentration. Cytotoxic activity was measured after incubation with target cells for 3 or 24 h. The data are presented as the mean ± SD of three independent experiments. Asterisks indicate that differences from the control are significant at *p* < 0.05 (two-way ANOVA).

The PBMC pool was treated with Hsp70 for six days, and activated lymphocytes were tested for cytotoxic activity by incubating them with target cells for 3 to 24 h. As follows from Figure 2A, Hsp70-activated lymphocytes showed cytotoxic activity against target cells at different time intervals, which could be explained by induction of different mechanisms of cytotoxicity at the corresponding intervals. Previously, it was shown that Tag7 binds to TREM-1 and induces appearance of cytotoxic lymphocytes [17]. Due to this fact, Tag7-activated lymphocytes were used as positive control. Both “slow” and “rapid” cytotoxicity dropped abruptly in the presence of LP17, a specific TREM-1 inhibitor, indicating that cytotoxic lymphocyte activation by Hsp70 is a TREM-1-dependent process.

Experiments with four cell lines used as target cells showed that Hsp70-activated cytotoxic lymphocytes killed only MHC-negative K562 and Molt4 cells [18,19], while cell lines expressing MHC-related antigens on their surface (HEK-293T and HeLa) proved to be insensitive to their effect (Figure 2B) [20,21].

Cytotoxicity of activated lymphocytes increased with an increase in Hsp70 concentration, with the half maximal effective concentration (EC_50_) being 0.5 nM (Figure 2C). Inhibition of cytotoxic activity by LP17 was also concentration-dependent: The half maximal inhibitory concentration (IC_50_) was 0.01 nM LP17 (Figure 2D). It should be noted that Hsp70-induced cytotoxicity was not completely suppressed even at a still higher concentration of the inhibitor (Figure 2A,D), suggesting that part of this cytotoxicity may be independent of TREM-1 activation.

Thus, Hsp70 binding to TREM-1 leads to activation of cytotoxic lymphocyte subpopulations capable of killing MHC-negative cells. To gain an insight into the mechanism of this activation, it was necessary to find out (1) what immune cells are involved in the activation of signal transduction, (2) what subpopulations of cytotoxic lymphocytes are activated by Hsp70, and (3) what are the mechanisms of toxic action of these subpopulations on tumor cells.

### 2.3. CD14+ Monocytes and CD4+ T-Lymphocytes Are Involved in Activation of Hsp70-Dependent Cytotoxicity

**Figure 3:**
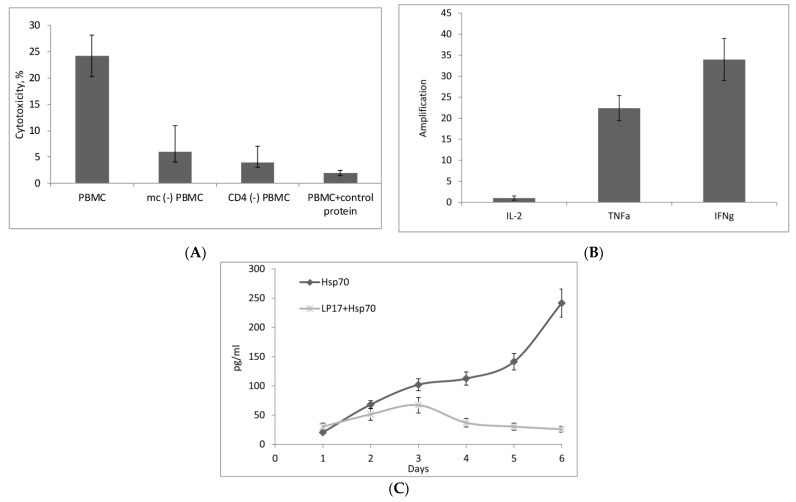
(**A**) Effect of CD14+ monocyte and CD3+CD4+ lymphocyte subpopulations on the formation of cytotoxic activity of Hsp70-activated PBMCs. These subpopulations were removed by magnetic separation, PBMCs were treated with Hsp70 for six days, and cytotoxicity of activated lymphocytes was measured after 24 h incubation with target cells. PBMCs incubated with control protein (BSA) were used as negative control. The data are presented as the mean ± SD of three independent experiments. (**B**) The levels of IL-2, TNFα, and IFNγ mRNAs measured by qPCR in monocytes incubated with Hsp70 for 3 h. (**C**) Dynamics of IL-2 secretion by PBMCs incubated with Hsp70 or LP17 + Hsp70 for six days.

Using magnetic separation, the PBMC pool was depleted of CD14+ monocytes or CD4+ T lymphocytes, treated with Hsp70 for six days, and the remaining PBMCs were tested for cytotoxicity (BSA (control protein) was used as negative control to exclude non-specific activation of PBMC). In both variants, their cytotoxic activity was found to be reduced (Figure 3A). These results indicated that the above immune cell subpopulations were involved in the transduction of the activation signal to the effector lymphocytes. The fact that Hsp70-induced cytotoxicity was not completely suppressed upon the removal of monocytes or CD4+ T-lymphocytes, confirms that Hsp70 can also cause TREM-1-independent activation of cytotoxic lymphocytes. It is well known that NK cells can be directly activated by Hsp70 [22]. We have established that, for the standard cytotoxic activity of purified NK cells, their ratio to target cells should not be less than 20:1 (Supplement 3). In mc(−) PBMCs, ratio of NK cells may be insufficient to achieve the standard cytotoxic activity against tumor cells. On the other hand, with an increase in the ratio of mc (-) PBMCs to the target cells, the cytotoxic activity also raises (Supplement 4).

As was shown in our recent study [17], monocytes and CD4+ T-lymphocytes are also involved in the activation of cytotoxic lymphocytes upon the interaction of TREM-1 with the innate immunity protein Tag7, with monocytes secreting cytokines TNFα and IFNγ, while CD4+ T-lymphocytes showing activation of *IL-2* gene expression followed by IL-2 secretion. Therefore, we tested how the expression of genes encoding these cytokines and their secretion change upon treatment with Hsp70.

CD14+ monocytes isolated from PBMC by magnetic separation were incubated with Hsp70 for 3 h and analyzed by qPCR for changes in the levels of mRNAs of the above three cytokines. As shown in Figure 3B, the levels of TNFα and IFNγ mRNAs in the monocytes increased upon their interaction with Hsp70, while the level of IL-2 mRNA remained very low.

We then studied the dynamics of IL-2 secretion by PBMCs incubated with Hsp70 for six days. Aliquots of the conditioned medium were taken every day to measure IL-2 concentration by ELISA method. Figure 3C shows that the medium conditioned by Hsp70-activated PBMCs contained IL-2, with its concentration reaching a peak by day six. Preliminary treatment of PBMCs with TREM-1 inhibitor LP17 resulted in a significant reduction of IL-2 level in the medium.

Thus, incubation with Hsp70 leads to activation of cytokine TNFα and IFNγ gene expression in monocytes and the onset of IL-2 secretion by PBMCs.

### 2.4. Hsp70 Stimulates Cytotoxic Activity of NK Cells, CD4+ T-Lymphocytes, and CD8+ T-Lymphocytes

**Figure 4:**
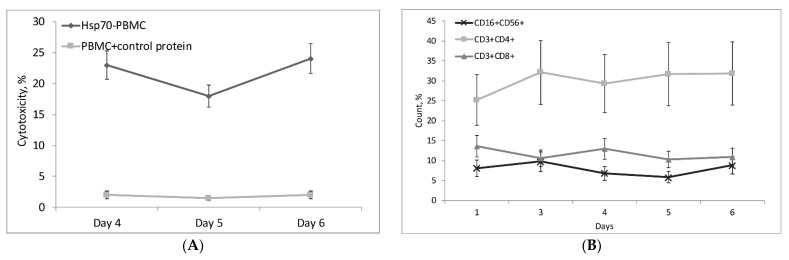

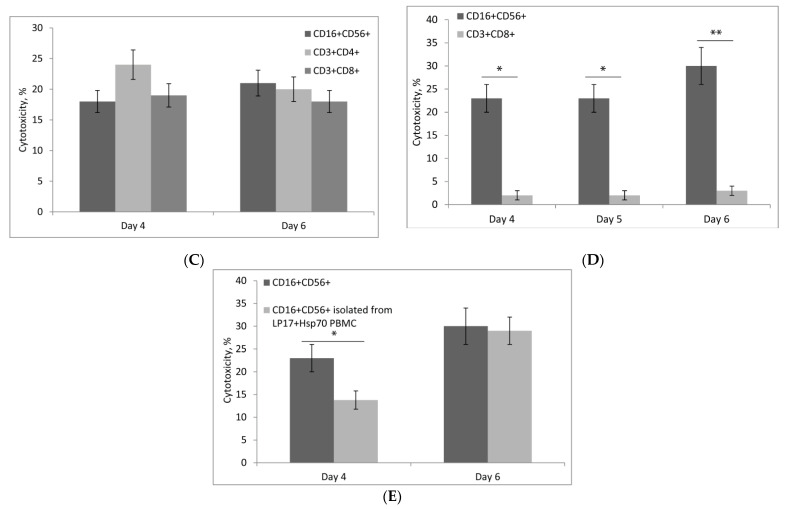
Characteristics of cytotoxic lymphocyte subpopulations activated by Hsp70 in PBMCs. (**A**) Cytotoxic activity of lymphocytes depending on the time of treatment with Hsp70. PBMCs incubated with control protein (BSA) were used as control. Lymphocytes were incubated with target cells for 24 h. (**B**) Changes in the ratio of CD16+CD56+, CD3+CD4+, and CD3+CD8+ lymphocyte subpopulations in PBMC depending on the time of treatment with Hsp70 (measured using flow cytometry). (**C**) Cytotoxic activity of lymphocyte subpopulations on days four and six of PBMC treatment with Hsp70. Lymphocytes were incubated with target cells for 24 h. (**D**) Cytotoxic activity of NK cells stimulated by Hsp70. NK cells and CD8+ T-Lymphocytes (control) were incubated with target cells for 3 h. (**E**) Cytotoxic activity of NK cells isolated from PBMC treated LP17 + Hsp70 or Hsp70 only on days four and six. Lymphocytes were incubated with target cells for 3 h. The data are presented as the mean ± SD of three independent experiments. Differences from the control in all cases are significant at * *p* < 0.05, at ** *p* < 0.03 (2-way ANOVA).

The next step was to find out what populations of cytotoxic lymphocytes are activated by Hsp70. Figure 4A shows the dependence of PBMC cytotoxic activity on the time of incubation with Hsp70. This activity manifested itself on day four, decreased slightly on day five, and increased again by day six. BSA (control protein) was used as negative control to exclude non-specific activation of PBMC (Figure 4A). Tests conducted with specific antibodies using flow cytometry showed that PBMCs included three cytotoxic lymphocyte subpopulations: CD16+CD56+, CD3+CD4+, and CD3+CD8+ (Figure 4B; primary data is presented in Supplement 5). Each subpopulation was isolated using magnetic separation and tested for cytotoxicity on days four and six, and all of them proved to be active (Figure 4C).

As shown previously, Hsp70 can activate the cytotoxic action of NK (CD16+CD56+) cells [22,23]. Therefore, it could be hypothesized that NK cell activation in PBMCs treated with Hsp70 may occur independently of TREM-1-dependent signaling. To test this hypothesis, NK cells were isolated from a fresh PBMC pool and incubated with Hsp70, measuring their cytotoxic activity on days four, five, and six. CD8+ T-Lymphocytes incubated with Hsp70 were used as control. Figure 4D shows that Hsp70 stimulated the cytotoxic activity of NK cells on days four to six, but had no such effect on CD8+ T-Lymphocytes. Thus, Hsp70 under our experimental conditions could induce TREM-1-independent cytotoxic activity developing as a result of direct Hsp70 action on NK cells.

We then tested the ability of Hsp70 to induce TREM-1-dependent cytotoxicity of NK cells. To this point, PBMCs were incubated with Hsp70 in the presence of TREM-1 inhibitor LP17 or without it for certain time to measure the cytotoxic activity of NK cells isolated on days four and six. The level of NK cytotoxicity induced by Hsp70 on day four was found to decrease by 40% when the Hsp70–TREM-1 interaction was blocked. There is no difference in cytotoxicity of NK on day six activated by Hsp70 or Hsp70+LP17 (Figure 4E). It appears that Hsp70 on day four activates both TREM-1-dependent and TREM-1-independent cytotoxicity of NK cells, but TREM-dependent activation ceases by day six.

### 2.5. Hsp70-Activated NK Cells Secrete Granzymes, While Hsp70-Activated CD8+ and CD4+ T-Lymphocytes Kill Tumor Cells via the FasL–Fas Interaction

PBMCs were incubated with Hsp70 to isolate NK cells on day 4 and CD8+ and CD4+ T-lymphocytes on day six. These subpopulations were tested for cytotoxicity, alone and in the presence of specific antibodies or inhibitors, to find out what molecules expressed on the cell surface are necessary for lymphocytes to recognize their target cells and what cytotoxic processes they induce in tumor cells.

**Figure 5:**
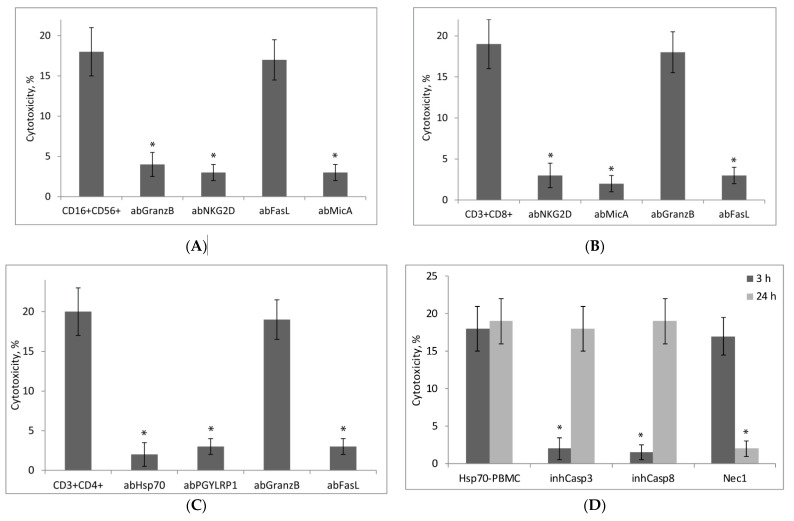
Experiments to evaluate the mechanism of cytotoxic action of PBMC subpopulations activated by Hsp70. Cytotoxicity against K562 cells was measured. Each subpopulation was tested alone and in the presence of specific antibodies and inhibitors added 1 h before incubation of target cells with effector lymphocytes. (**A**) NK cells (CD16+CD56+) and (**B**) CD3+CD8+ lymphocytes were tested in the presence of antibodies to granzyme B, FasL, NKG2D, and MicA; (**C**) CD3+CD4+ lymphocytes, in the presence of antibodies to granzyme B, FasL, Tag7 (PGYLRP1), and Hsp70; (**D**) Hsp70-activated PBMCs were tested for 3 and 24 h in the presence of inhibitors of caspase 3, caspase 8, and RIP1 kinase (Nec1). The data are presented as the mean ± SD of three independent experiments. Asterisks indicate that differences from the control are significant at * *p* < 0.03 (two-way ANOVA).

Specific antibodies to the NKG2D-receptor and non-canonical MicA antigen completely blocked the cytotoxic activity of Hsp70-activated NK cells and CD8+ T-lymphocytes (Figure 5A,B). Therefore, the NKG2D-receptor and MicA may be considered responsible for the interaction of these lymphocytes with MHC-negative tumor cells. According to our previous data, the Tag7 protein expressed on the surface of cytotoxic CD4+ T-Lymphocytes can interact with Hsp70 on the membranes of tumor cells [19,20]. Figure 5C shows that antibodies to Tag7 and Hsp70 completely suppressed the cytotoxic activity of Hsp70-dependent CD4+ T-Lymphocytes. Apparently, the interaction of these cells was also due to the formation of intercellular Tag7–Hsp70 complex.

The cytotoxic action of NK cells was dependent on the secretion of granzymes that induce apoptosis in tumor cells (Figure 5A). Since neither CD8+ nor CD4+ T-Lymphocytes produce granzymes, their cytotoxicity was not suppressed by anti-granzyme B antibodies (Figure 5B,C). The cytotoxic action of these lymphocytes was due to the interaction of FasL expressed on their membranes with Fas-receptor on tumor cells.

To characterize cytotoxic processes developing in tumor cells at different rates under the effect of Hsp70-activated lymphocytes, experiments with specific inhibitors of caspases and RIP1 kinase were performed. The results showed that tumor cells interacting with lymphocytes for 3 h subsequently die by apoptosis, while their interaction with lymphocytes for 24 h leads to the development of RIPK1-dependent necroptosis (Figure 5D).

## 3. Discussion

Two important observations were made in this study: (1) The major heat shock protein Hsp70 is a ligand for the innate immunity receptor TREM-1, and (2) the interaction of these proteins induces TREM-1-dependent signaling and results in activation of noncanonical cytotoxic lymphocytes acting against MHC-negative tumor cells.

Hsp70 is a well-studied multifunctional protein. It belongs to the chaperone family and is responsible for normal protein folding in the norm and under stress. Hsp70 can also have an effect on intracellular processes, in particular, by suppressing apoptosis [21,22], thereby contributing to the viability of cells (including tumor cells).

Identification of Hsp70 on tumor cell membranes has suggested new functions for this protein in the regulation of both innate and adaptive immunity [23]. It has been shown that membrane-bound Hsp70 serves as a tumor-specific antigen readily recognized by both NK cells [22,23,24,25,26,27] and cytotoxic CD4+CD25+ T-lymphocytes [20]. Moreover, Hsp70 is involved in the granzyme B-dependent cytolysis of tumor cells under the effect of NK cells [28].

It has also been found that viable tumor cells release Hsp70, both free and bound to exosomal lipids [29] and that the released Hsp70 can stimulate cytotoxic, proliferative, and migratory activities of NK cells [22,24,25,27,30]. Hsp70 in adaptive immunity stimulates presentation of antigens in complex with MHC1 and MHC2 proteins to cytotoxic CD8+ and helper CD4+ T-lymphocytes [24]. Moreover, Hsp70 is secreted by cytotoxic CD8+ T-Lymphocytes in complex with Tag7 protein [31,32,33], and this complex induces lysis of tumor cells carrying the TNFR1 receptor on their surface [34]. Hsp70 also induces the release of pro- and anti-inflammatory cytokines [35], secretion of IFNγ, and can perform a cytokine-like function [36].

We revealed a new role for Hsp70 in the regulation of adaptive immunity, by showing that Hsp70-dependent activation of TREM-1 receptor stimulates the appearance of non-canonical cytotoxic lymphocyte subpopulations that kill tumor cells evading immune control.

Our data also broaden the views on the mechanism of TREM-1-dependent signaling by showing that the crucial stages of activation signaling do not depend on the nature of inducer ligand. We have shown previously that the innate immunity protein Tag7 (PGLYRP1), recently described as a ligand for TREM-1, also induces activation of cytotoxic lymphocytes that kill MHC-negative tumor cells [16]. The interaction of Tag7 with TREM-1 in monocytes stimulates the expression of genes for TNFα and IFNγ and secretion of these cytokines followed by activation of CD4+T-Lymphocytes, which leads to the enhanced expression of *IL-2* gene and secretion of this cytokine, which plays a key role in the activation of cytotoxicity [17].

**Scheme 1:**
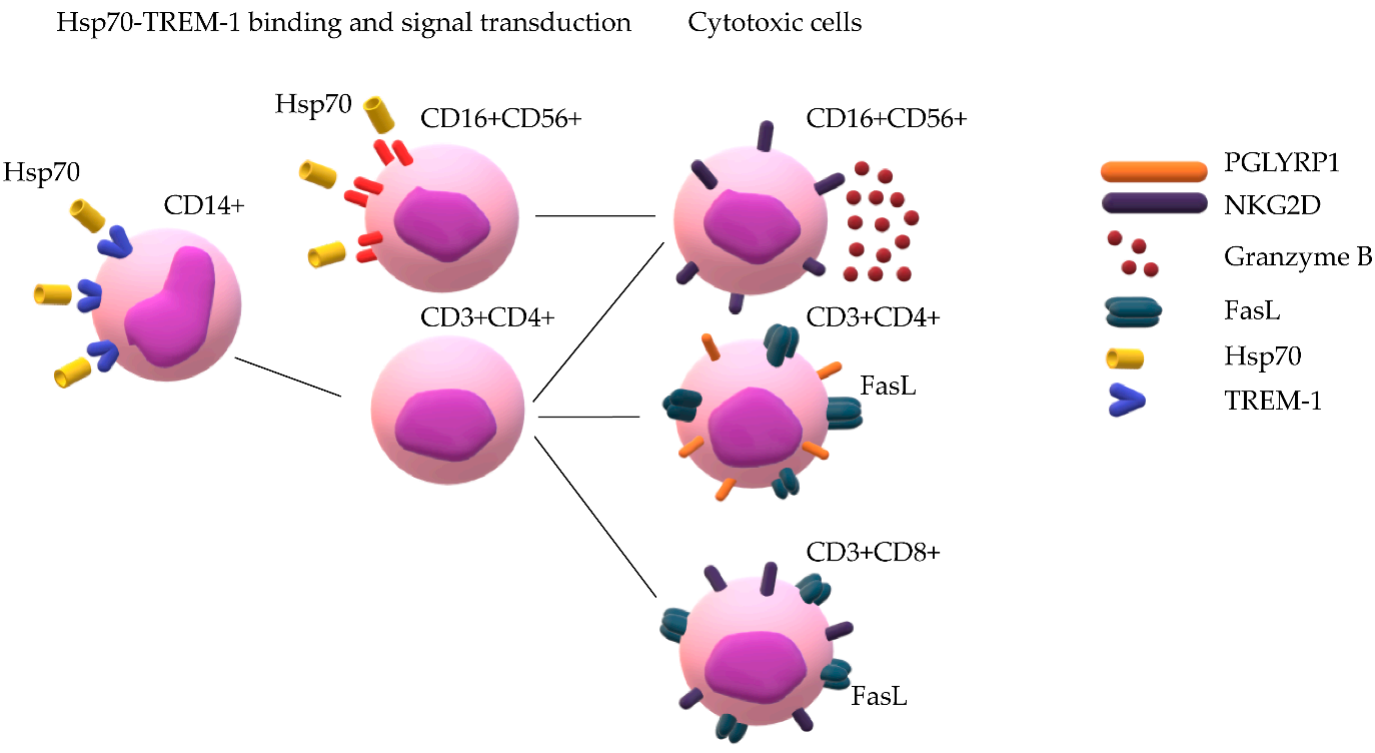
Hsp70 binds to TREM-1 on monocytes and induces mRNA expression of TNFα and IFNγ. Signal transduction recruits CD3+CD4+ lymphocytes and leads to appearance of IL-2 in conditioned medium, which promotes the maturation of cytotoxic lymphocytes CD3+CD4+, CD16+CD56+, and CD3+CD8+. At the same time, Hsp70 directly binds to NK cells and promotes their transition to cytotoxic cells.

We have shown here that the interaction of TREM-1 with another ligand, Hsp70, also leads to a sequential activation of monocytes and regulatory CD4+ T-Lymphocytes and to secretion of IL-2 that is necessary for the formation of cytotoxic lymphocytes. A hypothetical process of cytotoxic lymphocyte activation by Hsp70 appears to be as follows: Hsp70 binding to TREM-1 induces the expression of genes encoding TNFα and IFNγ; these cytokines, in turn, activate the expression of *IL-2* gene in CD4+ T-Lymphocytes and subsequent IL-2 secretion, which depends on TREM-1. At the next step, cytotoxic lymphocyte subpopulations involved in innate and adaptive immunity—NK cells and CD8+ and CD4+ T-Lymphocytes—are activated in PBMCs (Scheme 1).

As noted above, activation of NK cells by Hsp70 is a long established fact [25]. Here we showed that this activation is TREM-1-dependent.

The Hsp70-activated CD8+ and CD4+ T-Lymphocytes kill evasive tumor cells via the FasL–Fas interaction by inducing in them alternative cytotoxic processes, apoptosis and necroptosis. In the above case, these lymphocytes cause death by necroptosis even in those tumor cells where the pathway of apoptotic signal transduction is blocked, which is important in terms of antitumor defense.

We believe that the data presented in this study will expand knowledge of the role of Hsp70 in antitumor immunity.

## 4. Materials and Methods

### 4.1. Cell Culture and Sorting

K562 and Molt4 cells were cultured in RPMI-1640; HeLa and HEK-293T, in DMEM with 2 mM L-glutamine and 10% FCS (Invitrogen, Carlsbad, CA, USA). Human peripheral blood mononuclear cells (PBMCs) were isolated from the total leukocyte pool of healthy donors by Ficoll-Hypaque density gradient centrifugation, as described [33], and cultured at a density of 4 × 10^6^ cells/mL in RPMI-1640 with 10^–9^ M Hsp70 (unless otherwise specified), BSA (10^−9^ M) or Tag7 (10^−9^ M) for a certain time. Cell sorting was performed using CD16+CD56+, monocytes, CD8+, and CD4+ negative isolation magnetic bead kits, and sheep anti-rabbit Dynabeads (Dynal Biotech ASA, Norway) according to the manufacturer’s protocol.

### 4.2. Proteins and Antibodies

The cDNAs for the recombinant human Hsp70 were subcloned into pQE-31 and expressed in *Escherichia coli* M15 (pREP4) (Qiagen, Germantown, MD, USA). Hsp70 was purified as described [33]. The Pierce Chromogenic Endotoxin Quant Kit (Thermo Fisher Scientific, Waltham, MA, USA) detected no bacterial LPS in the recombinant Hsp70 preparation. Tag7 was produced as described [33], sTREM-1 was obtained according to [10]. BSA was from Sigma (Sigma–Aldrich, Burlington, CA, USA). Inhibitory peptide LP17 (LQVTDSGLYRCVIYHPP, 10^−9^ M) was added to lymphocytes 1 h before the Hsp70 treatment. Polyclonal antibodies to the Tag7 (PGLYRP1; RRID:AB_10960204), MicA (RRID:AB_10607089) were from Sigma (Sigma–Aldrich, Burlington, CA, USA); antibodies to the Hsp70 (AB_2866870) were from Invitrogen (Thermo Fisher Scientific, Waltham, Massachusetts, USA); antibodies to Granzyme B (RRID:AB_304251) and TREM-1 (RRID:AB_10864342) were from Abcam (Cambridge, UK); antibodies to NKG2D (RRID:AB_628024) and C-terminus of FasL (C-178; RRID:AB_2246664) were from Santa Cruz Biotechnology (Santa Cruz Biotechnology, Santa Cruz, CA, USA).

### 4.3. Affinity Chromatography, Immunoadsorption, and Immunoblotting

Two columns with CNBr-activated Sepharose 4B (GE Healthcare, Chicago, IL, USA) were prepared. Following the manufacturer’s protocol, one column was conjugated with sTREM-1 (aa 1–200, His-tag, antibodies-online GmbH, Germany) and the other with Hsp70. sTREM-1 was loaded onto the Hsp70-Sepharose column, and Hsp70 onto the sTREM-1-Sepharose column. The columns were thoroughly washed with PBS containing 0.5 M NaCl and PBS alone, and then eluted with 0.25 M triethylamine (TEA), pH 12. The eluted material was resolved by SDS-PAGE and detected with Coomassie blue in case of Hsp70, or blotted onto a nitrocellulose membrane in case of TREM-1. To detect TREM-1, primary rabbit anti-TREM-1 antibodies (1:1000, overnight) followed by secondary HRP-conjugated anti-rabbit antibody (GE Healthcare, Chicago, IL, USA; 1:15,000; 1 h) were used. The results were visualized using ECL Plus kit (GE Healthcare, Chicago, IL, USA) according to the manufacturer’s protocol. Monocytes (10^6^ cells) were incubated with Hsp70 (10^−8^ M) in the presence of BS^3^ (Thermo Fisher Scientific, Waltham, MA, USA), lysed in RIPA buffer (Sigma–Aldrich, Burlington, CA, USA) and purified using Dynabeads (M-280 Sheep Anti-Rabbit IgG; Dynal Biotech ASA, Norway) conjugated with anti-Hsp70 antibodies according to manufacturer’s protocol. This material was resolved by 10% PAGE followed by western blotting. To detect TREM-1 the protocol described above was used.

### 4.4. Cytotoxicity Assays

The K562 cells cultured in 96-well plates (6 × 10^4^ cells per well) were mixed with lymphocytes added at a 20:1 ratio and incubated at 37 °C in a 5% CO_2_ atmosphere for 3 to 24 h. The inhibition test was conducted with polyclonal antibodies (anti-NKG2D, anti-MicA, anti-Hsp70, anti-FasL, and anti-granzyme B) at a concentration of 20 μg/mL. Cytotoxic activity of lymphocytes was detected with a Cytotox 96 Assay kit (Promega, Madison, WI, USA) according to the manufacturer’s protocol. In the inhibition assays, the cells were initially treated for 1 h with the caspase 3 inhibitor Ac-DEVD-CHO, caspase 8 inhibitor Ac-IETD-CHO (50 μM each), or RIP1 kinase inhibitor necrostatin 1 (5 mM) (Sigma–Aldrich, Burlington, CA, USA), and then the lymphocytes were added.

### 4.5. Flow Cytometry

The cells were fixed with 1% paraformaldehyde (Sigma) and stained with appropriate antibodies at room temperature. CD8-TRITC (RRID:AB_10372207), CD3-FITC (RRID:AB_1470489), CD4-PE (RRID:AB_10376142), CD16-PE (RRID:AB_1464986), CD56-FITC (RRID:AB_10372519) antibodies were from Caltag Medsystems (Buckingham, UK). All antibodies were used at 1:200 ratio. The measurements were performed on a Cytomics FC 500 MPL flow cytometer (Beckman coulter, Brea, CA, USA), data were processed in EXPO32 software (Applied Cytometry Systems, Sheffield, UK, CXP Analysis 2.2).

### 4.6. ELISA

The levels of secretion of different cytokines were evaluated with Human IL-2, ELISA Kits (Thermo Fisher Scientific, Waltham, MA, USA) according to the manufacturer’s protocols.

### 4.7. qPCR

RNA was isolated from the fraction of monocytes (purified by magnetic cell separation) after their treatment with Hsp70 (10^–9^ M) for 3 h. Monocytes (2 × 10^6^ cells per sample) were lysed in 500 µL of Extract RNA Reagent (Eurogen, Russia) according to the manufacturer’s protocol. RNA measurement was conducted with NanoDrop (Thermo Fisher Scientific, Waltham, MA, USA) and equal amounts of RNA were used (1.1 µg). For detection of RNA degradation electrophoresis was used. The synthesis of cDNA was performed with oligo(dT) primers (Eurogen, Russia). The products were used for qPCR with primers for genes encoding RPLP0, TNFa, IL-2, and IFNγ. The level of RPLP0 mRNA was taken as a reference. The primers were as follows: for RPLP0: 5′-ACTGGAGACAAAGTGGGAGCC (forward), 5′-CAGACACTGGCAACATTGCG (reverse); for IFNγ: 5′ GGGTTCTCTTGGCTGTTACTG 3′ (forward), 5′ TTCTGTCACTCTCCTCTTTCCA 3′ (reverse); for TNFα 5′-CTTCTCCTTCCTGATCGTGC-3′ (forward), 5′-GCTGGTTATCTCTCAGCTCCA-3′ (reverse); IL-2 forward: 5′-AAACTCACCAGGATGCTCAC-3′, reverse: 5′-TGTTTCAGATCCCTTTAGTTCCAG-3′.

Measurements at each point were made in at least three replicates, and the mean value was calculated. Expression levels were quantified using the 2^ΔΔCt^ method.

### 4.8. Statistical Analysis

Data are presented as mean ± standard deviation. All experiments were repeated at least three times. Differences between treatment and control were tested for significance with MathCad software (Version 15.0, PTC, Cambridge, MA, USA) using Student’s *t*-test for experiments on cell treatment with a single agent and two-way ANOVA for experiments on cell treatment with two or more agents (see individual figure legends).

## Supplementary Materials

The following are available online at https://www.mdpi.com/article/10.3390/ijms22136889/s1.
