# Supplementary material for: Hsp70 Interacts with the TREM-1 Receptor Expressed on Monocytes and Thereby Stimulates Generation of Cytotoxic Lymphocytes Active against MHC-Negative Tumor Cells
Source: Int J Mol Sci. 2021 Jun 26;22(13):6889. doi: 10.3390/ijms22136889 (PMC8267615; doi:10.3390/ijms22136889)
Supplement: Supplementary file 1 [file ijms-22-06889-s001.zip › Supplementary materials 1-4, Fig1.pdf]

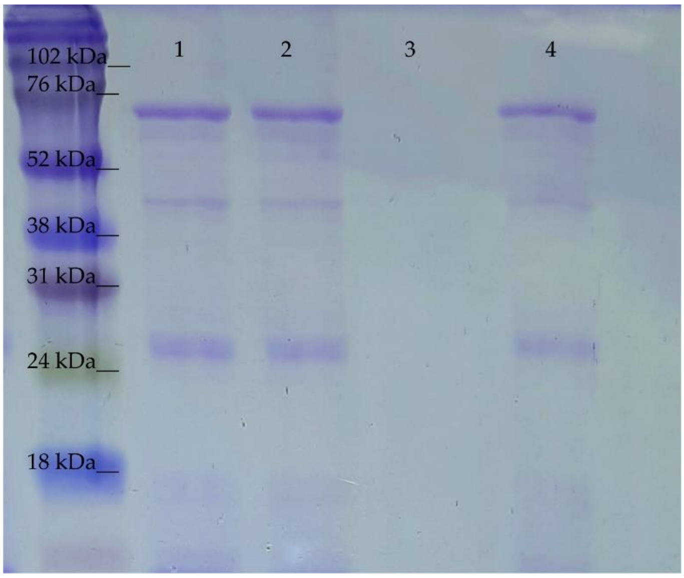

Figure 1A. (A) Hsp70 binding to sTREM-1 immobilized on a CNBr-Sepharose column (SDS-PAGE with Coomassie staining): (1) control Hsp70, (2) unbound fraction of Hsp70 loaded to column, (3) washed fraction from column before elution (4) Hsp70 eluted by TEA from the column.

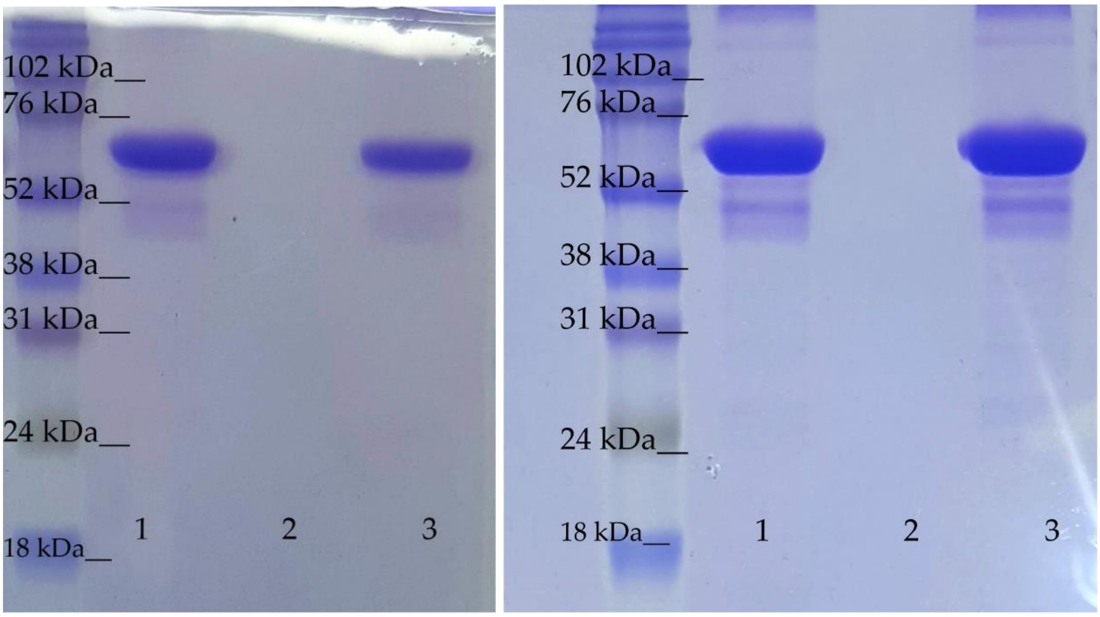

(A) (B)

Supplement 1 (A) BSA binding to sTREM-1 immobilized on a CNBr-Sepharose column (SDS-PAGE with Coomassie staining): (1) control BSA, (2) BSA eluted by TEA from the column (3) washed fraction from column before elution (B) BSA binding to Hsp70 immobilized on a CNBr-Sepharose column (SDS-PAGE with Coomassie staining): (1) control BSA, (2) BSA eluted by TEA from the column (3) washed fraction from column before elution.

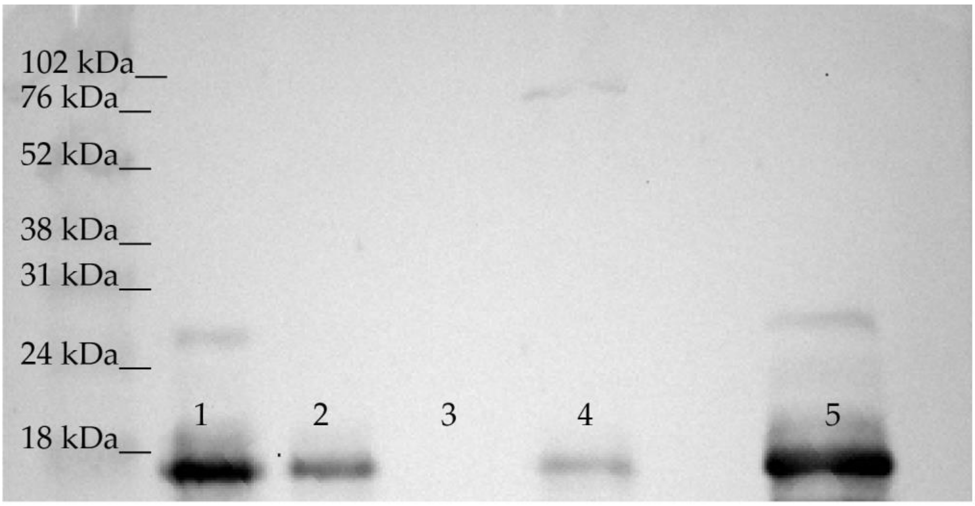

Figure 1B. TREM-1 binding with Hsp70 immobilized on a CNBr-Sepharose column (SDS-PAGE followed by Western blotting with specific anti-TREM-1 antibodies (1) Fraction washed with PBS, (2) Fraction washed with PBS+0.5M NaCl, (3) Fraction washed with PBS before elution, (4) TREM-1 eluted by TEA from the column, (5) control TREM-1.

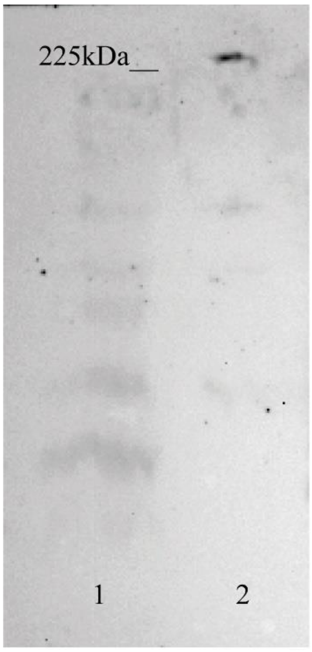

Figure 1C. Hsp70 binding to TREM-1 on the monocyte surface. Monocytes were incubated with Hsp70 in the presence of BS^3, lysed, and purified using Dynabeads conjugated with anti-Hsp70 antibodies. This material was resolved by 10% PAGE followed by Western blotting with anti-TREM-1 antibodies and visualized using the ECL plus detection Kit: (1) RPN 800E marker, (2) purified Hsp70–TREM-1 fraction.

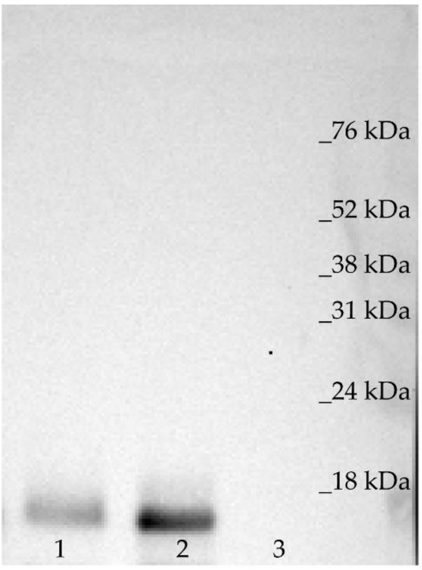

Supplement 2. Detection of sTREM-1 after purification of Hsp70-sTREM-1 complex with anti-Hsp70 coupled beads. sTREM-1 and Hsp70 incubated in PBS at 4^0C for an hour and sTREM-1 only purified with anti-Hsp70-coupled beads according to manufacturer's protocol. This material was resolved by 12% PAGE, followed by Western blotting with anti-TREM-1 antibodies, and visualized using the ECL plus detection Kit: (1) purified Hsp70–TREM-1 fraction, (2) sTREM-1 (control) (3) sTREM-1 only purified with anti-Hsp70 coupled beads.

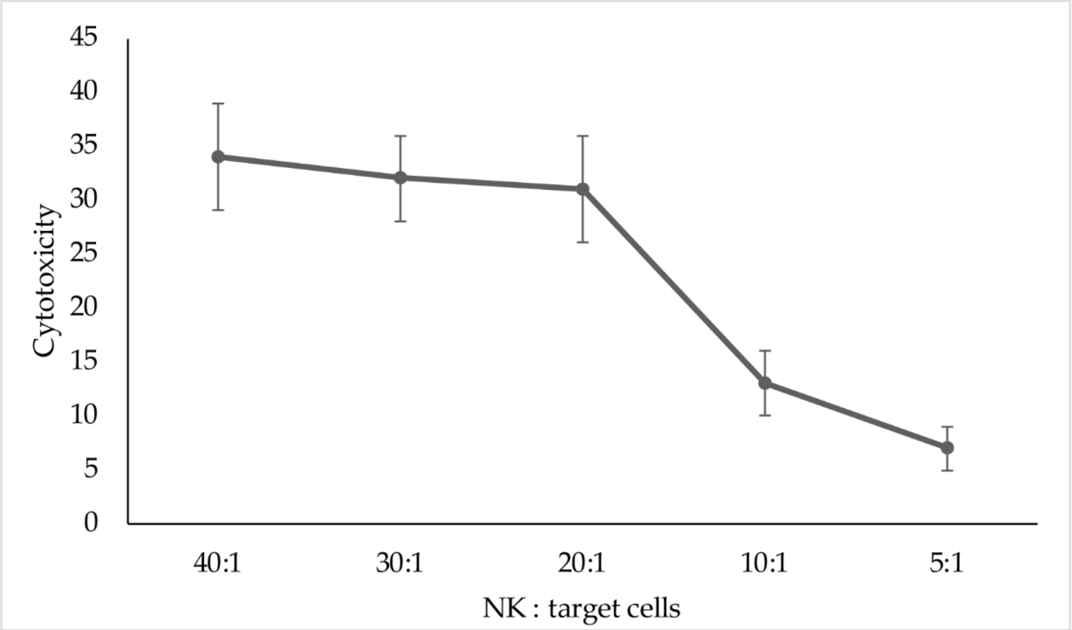

Supplement 3. Cytotoxic activity of NK, removed by magnetic separation from the 6-day Hsp70-activated PBMCs. Cytotoxic activity was measured after 3 h of incubation with target cells at different ratios. The data are presented as the mean ± SD of three independent experiments.

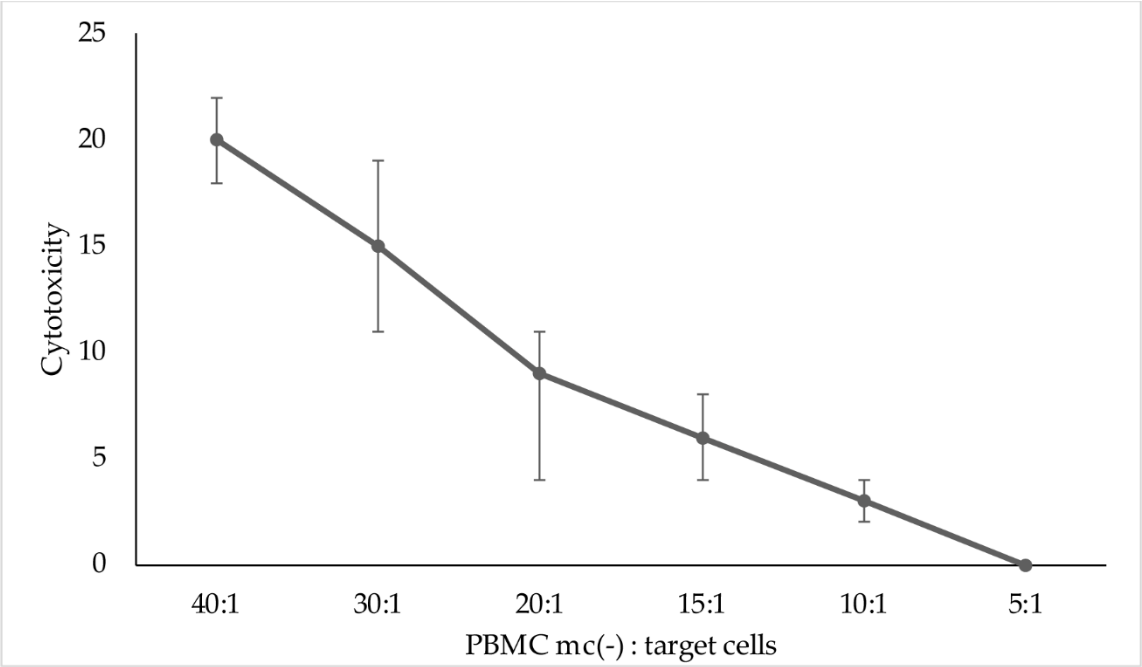

Supplement 4. Effect of CD14+ monocyte on the formation of cytotoxic activity of Hsp70-activated PBMCs. CD14+ monocyte subpopulations were removed by magnetic separation, PBMCs were treated with Hsp70 for 6 days, and cytotoxicity of activated lymphocytes was measured after 24-h incubation with target cells at different ratios. The data are presented as the mean ± SD of three independent experiments.
