# Supplementary material for: Hsp70 Interacts with the TREM-1 Receptor Expressed on Monocytes and Thereby Stimulates Generation of Cytotoxic Lymphocytes Active against MHC-Negative Tumor Cells
Source: Int J Mol Sci. 2021 Jun 26;22(13):6889. doi: 10.3390/ijms22136889 (PMC8267615; doi:10.3390/ijms22136889)
Supplement: Supplementary file 1 [file ijms-22-06889-s001.zip › Suppl5/Day 1 CD3-4-8.PDF]

3P Tanya lymph 240120.PRO : CXP

Institution: IBG
Protocol: 3P Tanya lymph 240120.PRO
Listmode Replay: New Protocol
Analysis Date: 20-Apr-2021, 19:58:36
Settings File: 3P Tanya lymph 240120.PRO, 24-Jan-2020, 19:38:03
Listmode File: 0day 1 donor1 CD3CD4CD8 00012752 2020-01-24 596.LMD

Run Date: 24-Jan-20, 19:38:21
Sample ID: 0day 1 donor1
User ID: Yashin
Acquisition Time/Events: 6.5s / 10000 (PROTOCOL)

Instrument SN: AK02006 Software Version: CXP 2.2

A] 0day 1 donor1 CD3CD4CD8 00012752 2020-01-24 596.L(F1)[A] 0day 1 donor1 CD3CD4CD8 00012752 2020-01-24 596.LMD : FL1 Log/FL4

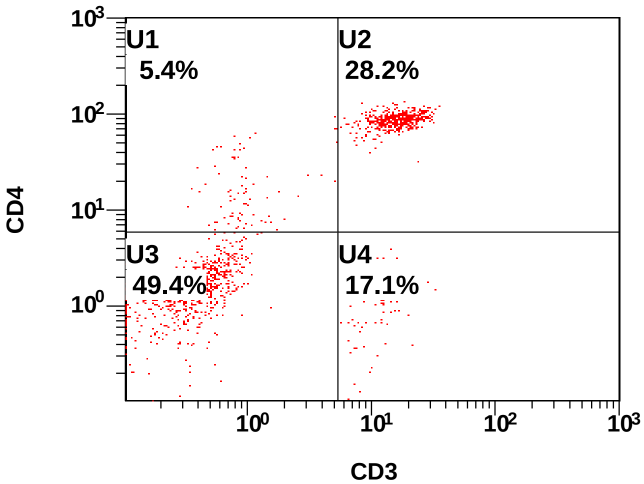

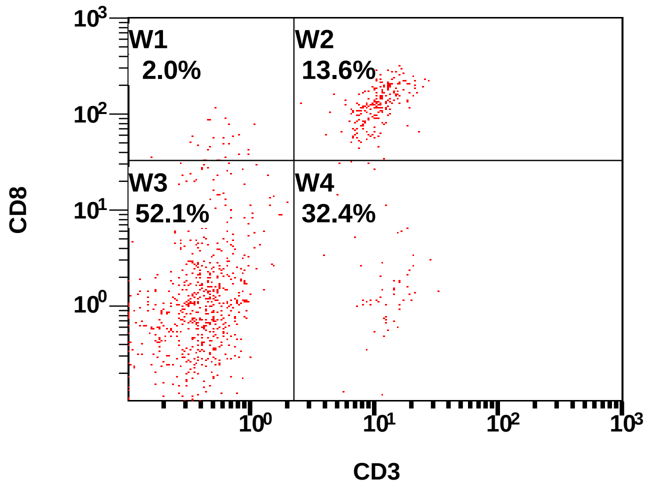

A] 0day 1 donor1 CD3CD4CD8 00012752 2020-01-24 596(F1)[Ungated] 0day 1 donor1 CD3CD4CD8 00012752 2020-01-24 596.LMD : SS Lin/l

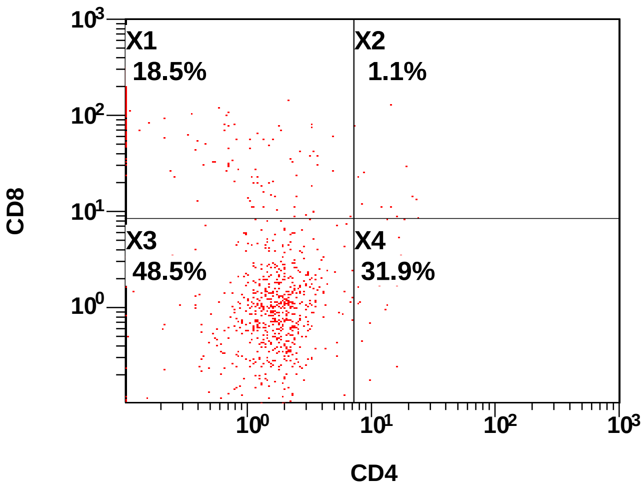

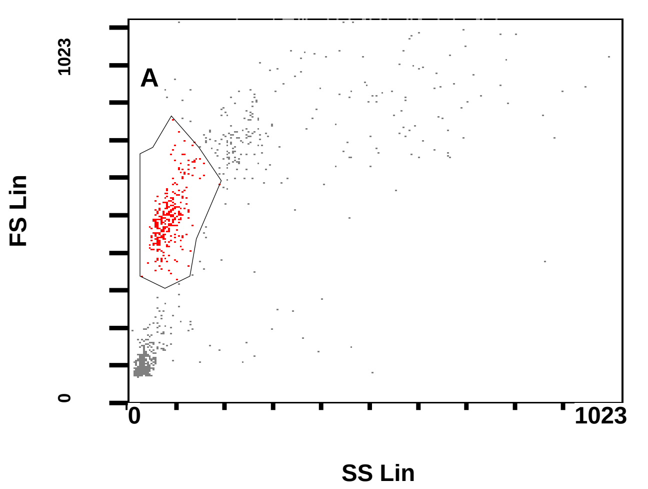

(F1)[A] 0day 1 donor1 CD3CD4CD8 00012752 2020-01-24 596.L(F1)[A] 0day 1 donor1 CD3CD4CD8 00012752 2020-01-24 596.LMD : FL2 Log

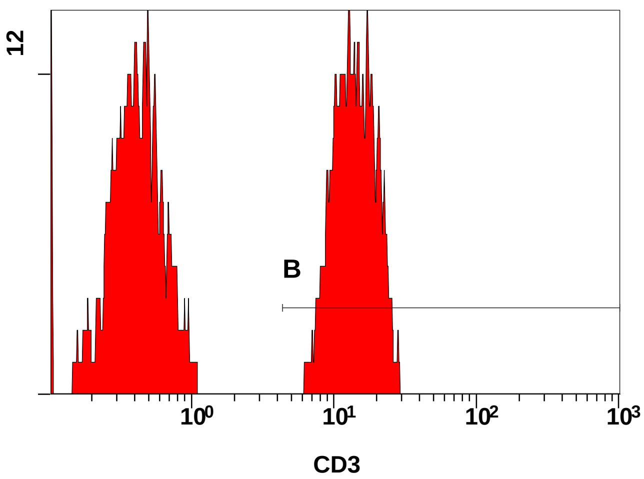

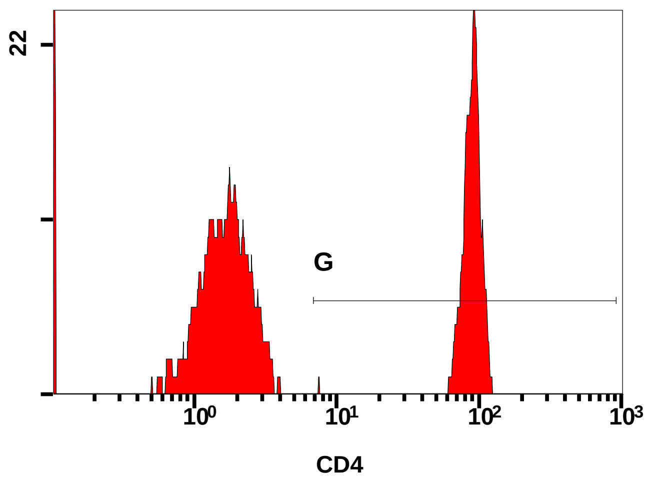

Institution: IBG
Protocol: 3P Tanya lymph 240120.PRO
Listmode Replay: New Protocol
Analysis Date: 20-Apr-2021, 19:58:37
Settings File: 3P Tanya lymph 240120.PRO, 24-Jan-2020, 19:38:03
Listmode File: 0day 1 donor1 CD3CD4CD8 00012752 2020-01-24 596.LMD

Run Date: 24-Jan-20, 19:38:21
Sample ID: 0day 1 donor1
User ID: Yashin
Acquisition Time/Events: 6.5s / 10000 (PROTOCOL)

Instrument SN: AK02006 Software Version: CXP 2.2

(F1)[A] 0day 1 donor1 CD3CD4CD8 00012752 2020-01-24 596.LMD : FL4 Log ·

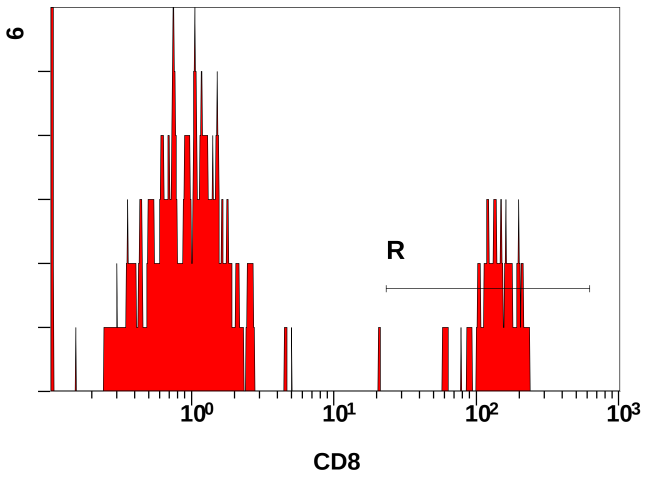

Statistical Analysis

PROGRAM INFORMATION

File:- 0day 1 donor1 CD3CD4CD8 00012752 2020-01-24 596.LMD

Gate:- A [A]

Compensation:- Advanced

Filename:- 0day 1 donor1 CD3CD4CD8 00012752 2020-01-24 596.LMD

Mean Calculation Method:-LOG-LOG

Region	Number	%Total	%Gated	X-Mean	Y-Mean
ALL	3164	31.64	100.00	6.95	27.2
ALL	3164	31.64	100.00	27.2	22.1
ALL	3164	31.64	100.00	6.95	22.1
ALL	3164	31.64	100.00	22.1	###
ALL	3164	31.64	100.00	27.2	###
ALL	3164	31.64	100.00	6.95	###
B	1443	14.43	45.61	14.7	###
G	1049	10.49	33.15	79.3	###
R	524	5.24	16.56	127	###
U1	170	1.70	5.37	1.16	21.9
U2	891	8.91	28.16	16.4	89.2
U3	1563	15.63	49.40	0.43	1.73
U4	540	5.40	17.07	12	0.487
W1	62	0.62	1.96	0.692	58.8
W2	430	4.30	13.59	11.4	145
W3	1647	16.47	52.05	0.442	2.13
W4	1025	10.25	32.40	15.9	0.602
X1	585	5.85	18.49	0.583	114
X2	36	0.36	1.14	18.2	33.7
X3	1536	15.36	48.55	1.78	1.09
X4	1009	10.09	31.89	81.7	0.192

File:- 0day 1 donor1 CD3CD4CD8 00012752 2020-01-24 596.LMD

Gate:- Ungated

Compensation:- Advanced

Filename:- 0day 1 donor1 CD3CD4CD8 00012752 2020-01-24 596.LMD

Mean Calculation Method:-LOG-LOG

Region	Number	%Total	%Gated	X-Mean	Y-Mean
ALL	10000	100.00	100.00	146	419
A	3164	31.64	31.64	85.5	503
