# Supplementary material for: Hsp70 Interacts with the TREM-1 Receptor Expressed on Monocytes and Thereby Stimulates Generation of Cytotoxic Lymphocytes Active against MHC-Negative Tumor Cells
Source: Int J Mol Sci. 2021 Jun 26;22(13):6889. doi: 10.3390/ijms22136889 (PMC8267615; doi:10.3390/ijms22136889)
Supplement: Supplementary file 1 [file ijms-22-06889-s001.zip › Suppl5/Day 3 CD16CD56.PDF]

Institution: IBG
Protocol: 3P Tanya lymph 240120.PRO
Listmode Replay: New Protocol
Analysis Date: 26-Apr-2021, 16:42:15
Settings File: 3P Tanya lymph 240120.PRO, 27-Jan-2020, 17:20:32
Listmode File: 3 day Hsp70-1d CD16 CD56 00012754 2020-01-27 598.LMD

Run Date: 27-Jan-20, 17:20:49
Sample ID: 3 day Hsp70-1d
User ID: Yashin
Acquisition Time/Events: 9.9s / 10000 (PROTOCOL)
Instrument SN: AK02006 Software Version: CXP 2.2

A] 3 day Hsp70-1d CD16 CD56 00012754 2020-01-27 598(F1)[Ungated] 3 day Hsp70-1d CD16 CD56 00012754 2020-01-27 598.LMD : SS Lin/I

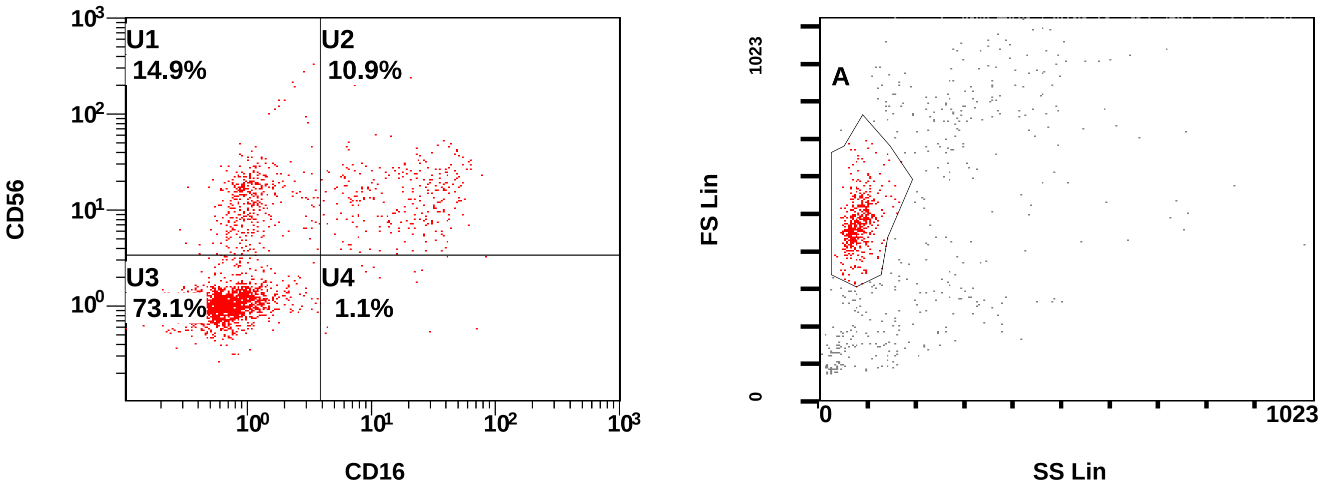

(F1)[A] 3 day Hsp70-1d CD16 CD56 00012754 2020-01-27 598.L(F1)[A] 3 day Hsp70-1d CD16 CD56 00012754 2020-01-27 598.LMD : FL2 Log

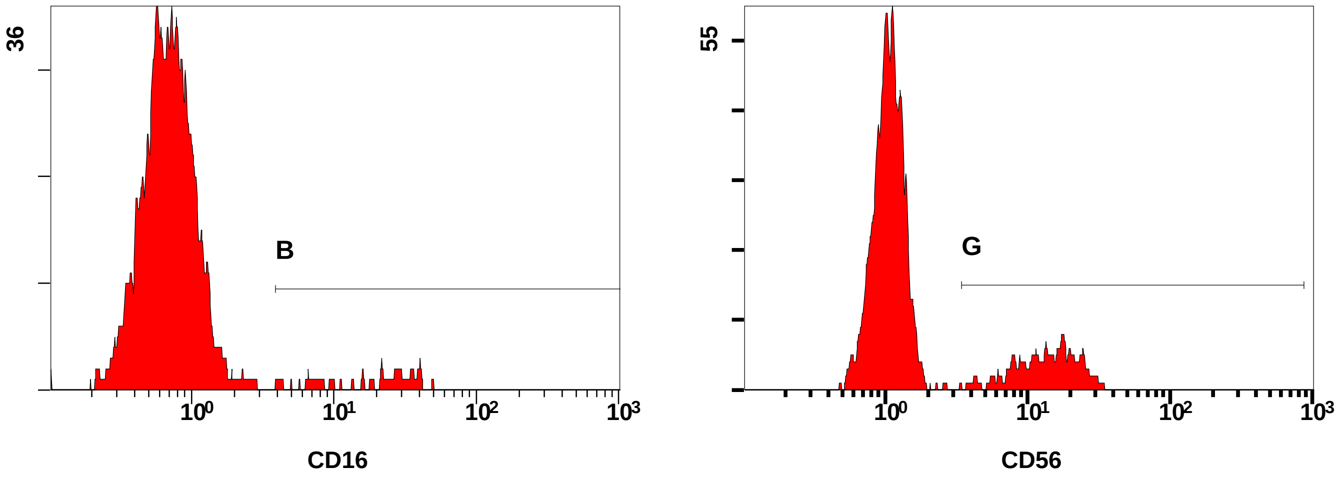

Statistical Analysis

PROGRAM INFORMATION

File:- 3 day Hsp70-1d CD16 CD56 00012754 2020-01-27 598.LMD

Gate:- A [A]

Compensation:- Advanced

Filename:- 3 day Hsp70-1d CD16 CD56 00012754 2020-01-27 598.LMD

Mean Calculation Method:-LOG-LOG

Region	Number	%Total	%Gated	X-Mean	Y-Mean
ALL	5089	50.89	100.00	3.37	6.07
ALL	5089	50.89	100.00	6.07	###
ALL	5089	50.89	100.00	3.37	###
B	610	6.10	11.99	22.3	###
G	1311	13.11	25.76	20.4	###
U1	759	7.59	14.91	1.16	20.2
U2	556	5.56	10.93	23	20.5
U3	3720	37.20	73.10	0.71	1.1
U4	54	0.54	1.06	15.4	1.54

File:- 3 day Hsp70-1d CD16 CD56 00012754 2020-01-27 598.LMD

Gate:- Ungated

Compensation:- Advanced

Filename:- 3 day Hsp70-1d CD16 CD56 00012754 2020-01-27 598.LMD

Mean Calculation Method:-LOG-LOG

Region	Number	%Total	%Gated	X-Mean	Y-Mean
ALL	10000	100.00	100.00	193	519
A	5089	50.89	50.89	81.2	483
