# Supplementary material for: Hsp70 Interacts with the TREM-1 Receptor Expressed on Monocytes and Thereby Stimulates Generation of Cytotoxic Lymphocytes Active against MHC-Negative Tumor Cells
Source: Int J Mol Sci. 2021 Jun 26;22(13):6889. doi: 10.3390/ijms22136889 (PMC8267615; doi:10.3390/ijms22136889)
Supplement: Supplementary file 1 [file ijms-22-06889-s001.zip › Suppl5/Day 4 CD16CD56.PDF]

Institution: IBG
Protocol: 3P Tanya lymph 240120.PRO
Listmode Replay: New Protocol
Analysis Date: 20-Apr-2021, 20:07:26
Settings File: 3P Tanya lymph 240120.PRO, 28-Jan-2020, 15:31:52
Listmode File: 4 day Hsp70 1d CD16 CD56 00012760 2020-01-28 604.LMD

Run Date: 28-Jan-20, 15:32:09
Sample ID: 4 day Hsp70 1d
User ID: Yashin
Acquisition Time/Events: 10.5s / 10000 (PROTOCOL)
Instrument SN: AK02006 Software Version: CXP 2.2

A] 4 day Hsp70 1d CD16 CD56 00012760 2020-01-28 604(F1)[Ungated] 4 day Hsp70 1d CD16 CD56 00012760 2020-01-28 604.LMD : SS Lin/l

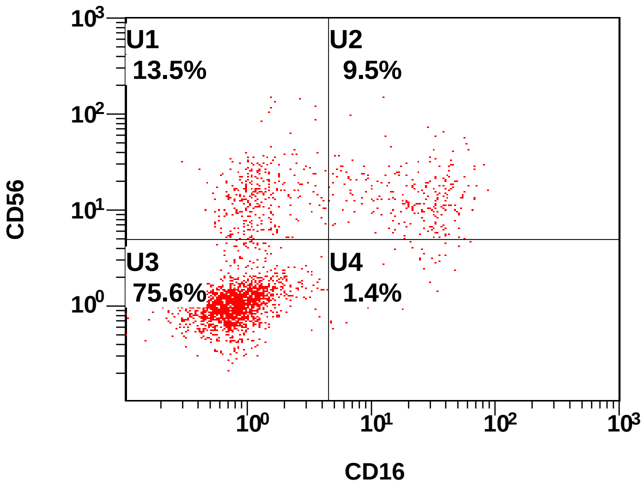

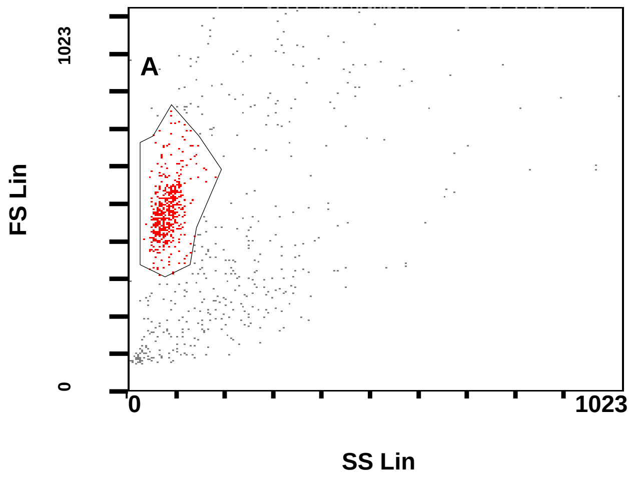

[F1)[A] 4 day Hsp70 1d CD16 CD56 00012760 2020-01-28 604.L(F1)[A] 4 day Hsp70 1d CD16 CD56 00012760 2020-01-28 604.LMD : FL2 Log

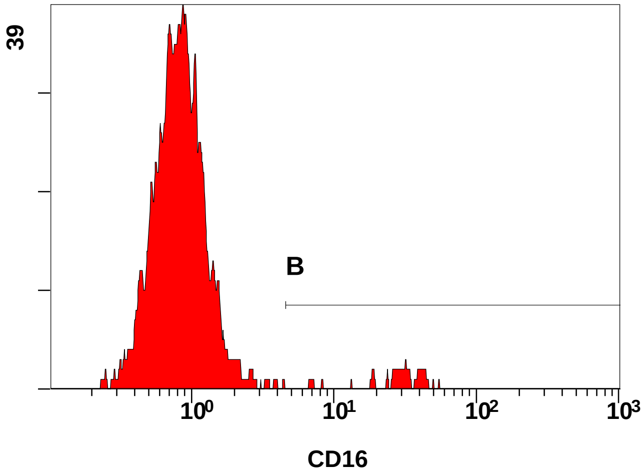

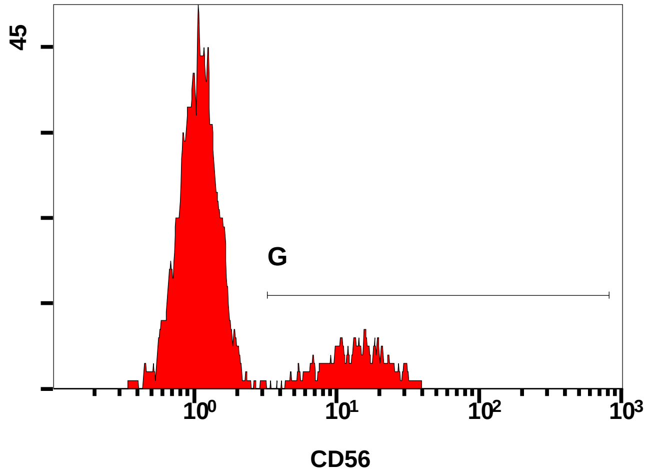

Statistical Analysis

PROGRAM INFORMATION

File:- 4 day Hsp70 1d CD16 CD56 00012760 2020-01-28 604.LMD					
Gate:- A [A]					
Compensation:- Advanced					
Filename:- 4 day Hsp70 1d CD16 CD56 00012760 2020-01-28 604.LMD					
Mean Calculation Method:-LOG-LOG					
Region	Number	%Total	%Gated	X-Mean	Y-Mean
ALL	5014	50.14	100.00	3.69	5.47
ALL	5014	50.14	100.00	3.69	###
ALL	5014	50.14	100.00	5.47	###
B	542	5.42	10.81	26.3	###
G	1240	12.40	24.73	18.7	###
U1	678	6.78	13.52	1.39	19.5
U2	474	4.74	9.45	27.2	20.2
U3	3793	37.93	75.65	0.857	1.17
U4	69	0.69	1.38	20.3	2.41

File:- 4 day Hsp70 1d CD16 CD56 00012760 2020-01-28 604.LMD					
Gate:- Ungated					
Compensation:- Advanced					
Filename:- 4 day Hsp70 1d CD16 CD56 00012760 2020-01-28 604.LMD					
Mean Calculation Method:-LOG-LOG					
Region	Number	%Total	%Gated	X-Mean	Y-Mean
ALL	10000	100.00	100.00	196	496
A	5014	50.14	50.14	79.1	481
