# Supplementary material for: Hsp70 Interacts with the TREM-1 Receptor Expressed on Monocytes and Thereby Stimulates Generation of Cytotoxic Lymphocytes Active against MHC-Negative Tumor Cells
Source: Int J Mol Sci. 2021 Jun 26;22(13):6889. doi: 10.3390/ijms22136889 (PMC8267615; doi:10.3390/ijms22136889)
Supplement: Supplementary file 1 [file ijms-22-06889-s001.zip › Suppl5/Day 4 CD3 -4-8.PDF]

3P Tanya lymph 240120.PRO : CXP

Institution: IBG
Protocol: 3P Tanya lymph 240120.PRO
Listmode Replay: New Protocol
Analysis Date: 20-Apr-2021, 20:04:34
Settings File: 3P Tanya lymph 240120.PRO, 28-Jan-2020, 15:34:51
Listmode File: 4 day Hsp70 1d CD3 CD4 CD8 00012761 2020-01-28 605.LMD

Run Date: 28-Jan-20, 15:35:08
Sample ID: 4 day Hsp70 1d
User ID: Yashin
Acquisition Time/Events: 9.3s / 10000 (PROTOCOL)

Instrument SN: AK02006 Software Version: CXP 2.2

A] 4 day Hsp70 1d CD3 CD4 CD8 00012761 2020-01-28 60!(F1)[A] 4 day Hsp70 1d CD3 CD4 CD8 00012761 2020-01-28 605.LMD : FL1 Log/FL

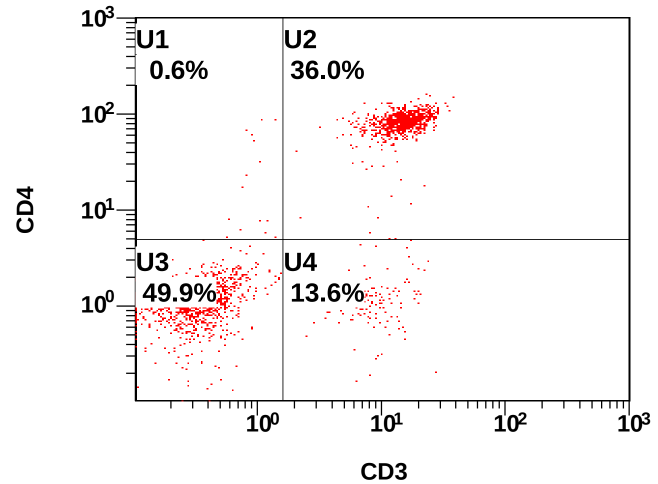

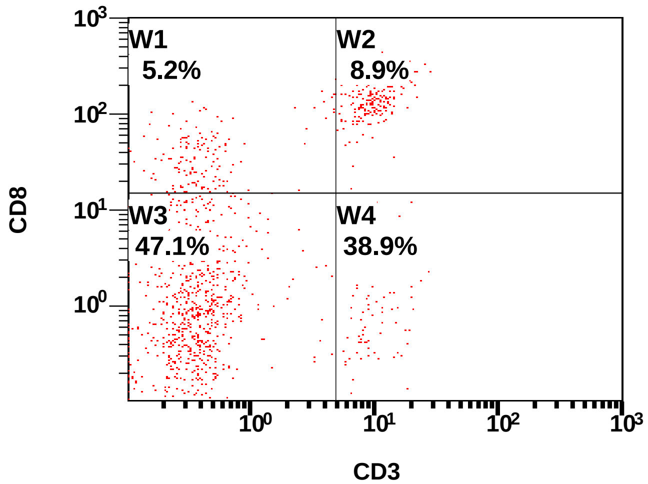

A] 4 day Hsp70 1d CD3 CD4 CD8 00012761 2020-01-28 6(F1)[Ungated] 4 day Hsp70 1d CD3 CD4 CD8 00012761 2020-01-28 605.LMD : SS Lin

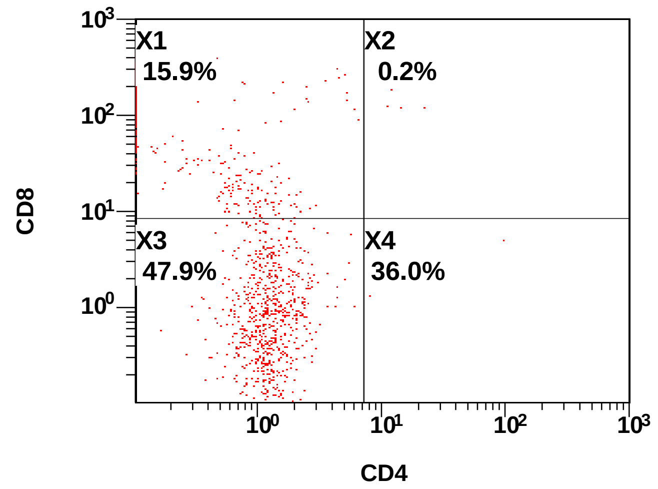

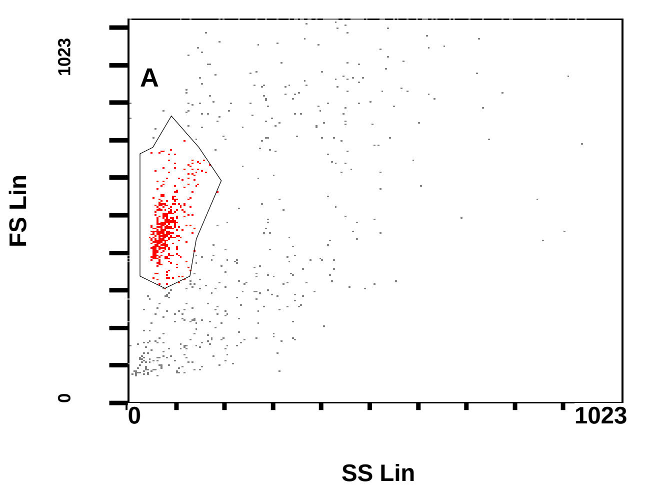

F1)[A] 4 day Hsp70 1d CD3 CD4 CD8 00012761 2020-01-28 60!(F1)[A] 4 day Hsp70 1d CD3 CD4 CD8 00012761 2020-01-28 605.LMD : FL2 Log

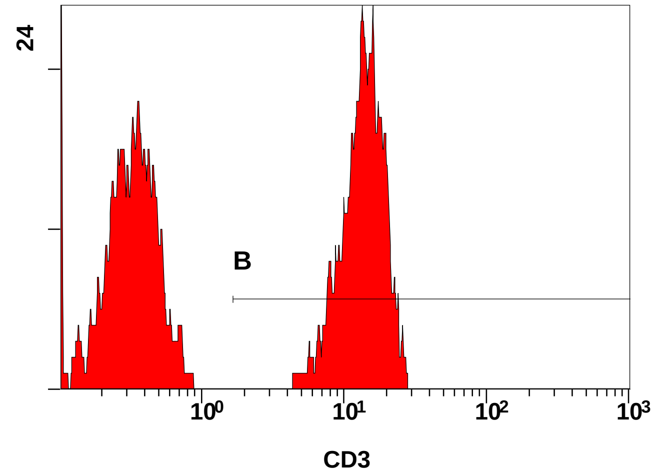

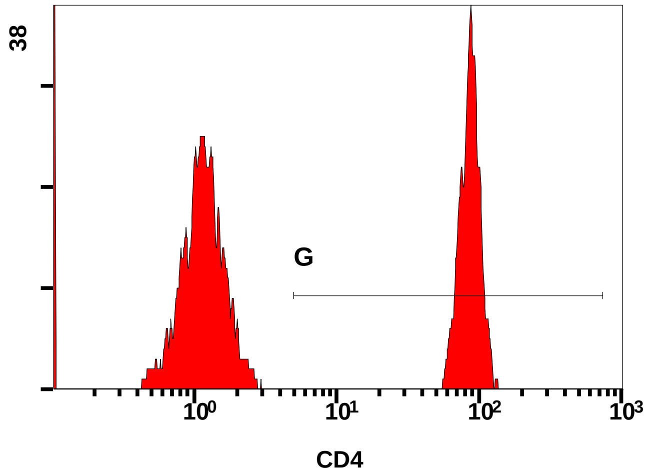

Institution: IBG
Protocol: 3P Tanya lymph 240120.PRO
Listmode Replay: New Protocol
Analysis Date: 20-Apr-2021, 20:04:35
Settings File: 3P Tanya lymph 240120.PRO, 28-Jan-2020, 15:34:51
Listmode File: 4 day Hsp70 1d CD3 CD4 CD8 00012761 2020-01-28 605.LMD

Run Date: 28-Jan-20, 15:35:08
Sample ID: 4 day Hsp70 1d
User ID: Yashin
Acquisition Time/Events: 9.3s / 10000 (PROTOCOL)

Instrument SN: AK02006 Software Version: CXP 2.2

:1)[A] 4 day Hsp70 1d CD3 CD4 CD8 00012761 2020-01-28 605.LMD : FL4 Log ·

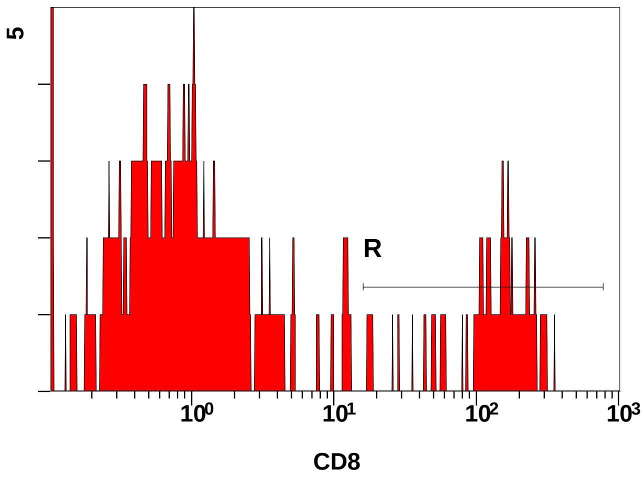

Statistical Analysis

PROGRAM INFORMATION

File:- 4 day Hsp70 1d CD3 CD4 CD8 00012761 2020-01-28 605.LMD

Gate:- A [A]

Compensation:- Advanced

Filename:- 4 day Hsp70 1d CD3 CD4 CD8 00012761 2020-01-28 605.LMD

Mean Calculation Method:-LOG-LOG

Region	Number	%Total	%Gated	X-Mean	Y-Mean
ALL	4697	46.97	100.00	7	31.7
ALL	4697	46.97	100.00	31.7	###
ALL	4697	46.97	100.00	7	###
ALL	4697	46.97	100.00	7	19.5
ALL	4697	46.97	100.00	19.5	###
ALL	4697	46.97	100.00	31.7	19.5
B	2327	23.27	49.54	13.8	###
G	1715	17.15	36.51	85	###
R	650	6.50	13.84	135	###
U1	26	0.26	0.55	1.03	45.3
U2	1689	16.89	35.96	15.2	85.6
U3	2343	23.43	49.88	0.354	1.17
U4	639	6.39	13.60	10	0.566
W1	242	2.42	5.15	0.816	50.6
W2	416	4.16	8.86	11.1	183
W3	2213	22.13	47.12	0.433	1.42
W4	1826	18.26	38.88	14.9	0.196
X1	747	7.47	15.90	0.492	117
X2	10	0.10	0.21	17.6	160
X3	2249	22.49	47.88	1.25	0.961
X4	1691	16.91	36.00	86	0.114

File:- 4 day Hsp70 1d CD3 CD4 CD8 00012761 2020-01-28 605.LMD

Gate:- Ungated

Compensation:- Advanced

Filename:- 4 day Hsp70 1d CD3 CD4 CD8 00012761 2020-01-28 605.LMD

Mean Calculation Method:-LOG-LOG

Region	Number	%Total	%Gated	X-Mean	Y-Mean
ALL	10000	100.00	100.00	200	507
A	4697	46.97	46.97	78.6	471
