# Supplementary material for: Hsp70 Interacts with the TREM-1 Receptor Expressed on Monocytes and Thereby Stimulates Generation of Cytotoxic Lymphocytes Active against MHC-Negative Tumor Cells
Source: Int J Mol Sci. 2021 Jun 26;22(13):6889. doi: 10.3390/ijms22136889 (PMC8267615; doi:10.3390/ijms22136889)
Supplement: Supplementary file 1 [file ijms-22-06889-s001.zip › Suppl5/Day 5 CD16CD56.PDF]

3P Tanya lymph 240120.PRO : CXP

Institution: IBG
Protocol: 3P Tanya lymph 240120.PRO
Listmode Replay: New Protocol
Analysis Date: 20-Apr-2021, 20:07:59
Settings File: 3P Tanya lymph 240120.PRO, 29-Jan-2020, 15:59:19
Listmode File: 5 day Hsp701d CD16 CD56 00012768 2020-01-29 612.LMD

Run Date: 29-Jan-20, 15:59:36
Sample ID: 5 day Hsp701d
User ID: Yashin
Acquisition Time/Events: 13.7s / 10000 (PROTOCOL)

Instrument SN: AK02006 Software Version: CXP 2.2

[A] 5 day Hsp701d CD16 CD56 00012768 2020-01-29 612.(F1)[Ungated] 5 day Hsp701d CD16 CD56 00012768 2020-01-29 612.LMD : SS Lin/F

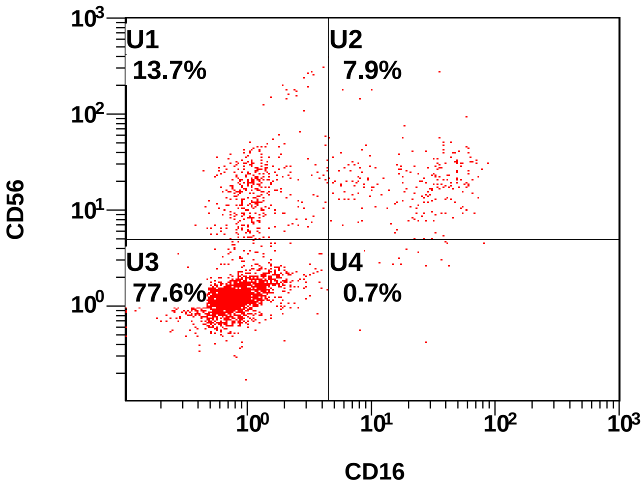

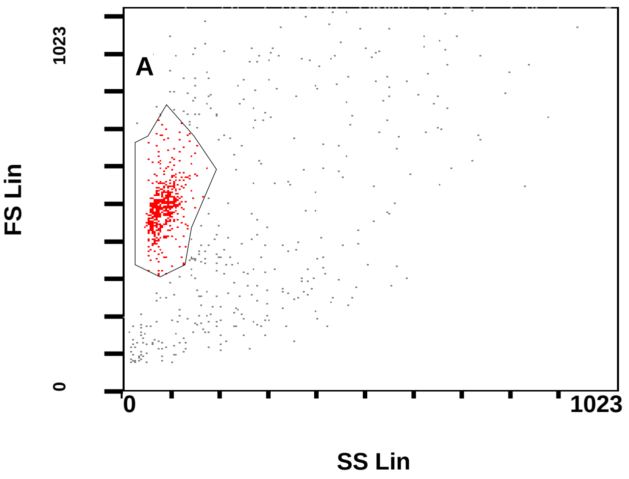

(F1)[A] 5 day Hsp701d CD16 CD56 00012768 2020-01-29 612.L(F1)[A] 5 day Hsp701d CD16 CD56 00012768 2020-01-29 612.LMD : FL2 Log

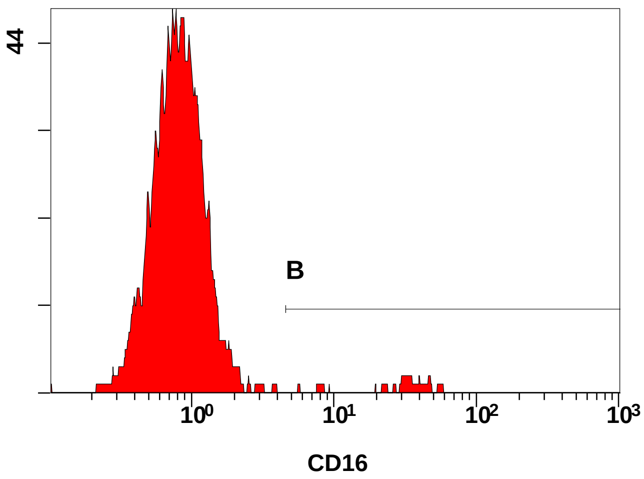

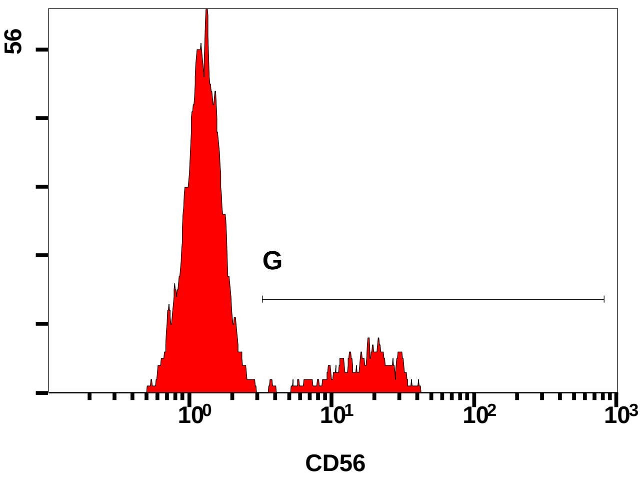

Statistical Analysis

PROGRAM INFORMATION

File:- 5 day Hsp701d CD16 CD56 00012768 2020-01-29 612.LMD					
Gate:- A [A]					
Compensation:- Advanced					
Filename:- 5 day Hsp701d CD16 CD56 00012768 2020-01-29 612.LMD					
Mean Calculation Method:-LOG-LOG					
Region	Number	%Total	%Gated	X-Mean	Y-Mean
ALL	5763	57.63	100.00	3.24	6.76
ALL	5763	57.63	100.00	3.24	###
ALL	5763	57.63	100.00	6.76	###
B	499	4.99	8.66	27.9	###
G	1328	13.28	23.04	25	###
U1	789	7.89	13.69	1.3	25.2
U2	458	4.58	7.95	28.6	28.4
U3	4473	44.73	77.62	0.835	1.34
U4	43	0.43	0.75	19	2.63

File:- 5 day Hsp701d CD16 CD56 00012768 2020-01-29 612.LMD					
Gate:- Ungated					
Compensation:- Advanced					
Filename:- 5 day Hsp701d CD16 CD56 00012768 2020-01-29 612.LMD					
Mean Calculation Method:-LOG-LOG					
Region	Number	%Total	%Gated	X-Mean	Y-Mean
ALL	10000	100.00	100.00	188	525
A	5763	57.63	57.63	82.4	496
