# Supplementary material for: Hsp70 Interacts with the TREM-1 Receptor Expressed on Monocytes and Thereby Stimulates Generation of Cytotoxic Lymphocytes Active against MHC-Negative Tumor Cells
Source: Int J Mol Sci. 2021 Jun 26;22(13):6889. doi: 10.3390/ijms22136889 (PMC8267615; doi:10.3390/ijms22136889)
Supplement: Supplementary file 1 [file ijms-22-06889-s001.zip › Suppl5/Day 5 CD3 -4-8.PDF]

3P Tanya lymph 240120.PRO : CXP

Institution: IBG
Protocol: 3P Tanya lymph 240120.PRO
Listmode Replay: New Protocol
Analysis Date: 20-Apr-2021, 20:04:59
Settings File: 3P Tanya lymph 240120.PRO, 29-Jan-2020, 16:01:52
Listmode File: 5 day Hsp70 1d CD3 CD4 CD8 00012769 2020-01-29 613.LMD

Run Date: 29-Jan-20, 16:02:09
Sample ID: 5 day Hsp70 1d
User ID: Yashin
Acquisition Time/Events: 12.6s / 10000 (PROTOCOL)

Instrument SN: AK02006 Software Version: CXP 2.2

\] 5 day Hsp70 1d CD3 CD4 CD8 00012769 2020-01-29 61:(F1)[A] 5 day Hsp70 1d CD3 CD4 CD8 00012769 2020-01-29 613.LMD : FL1 Log/FL

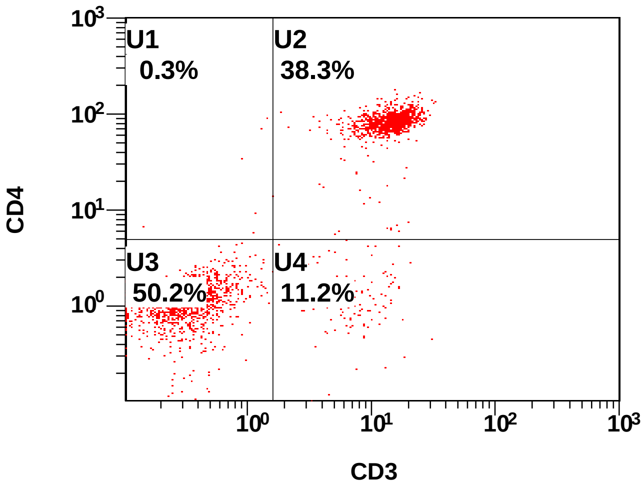

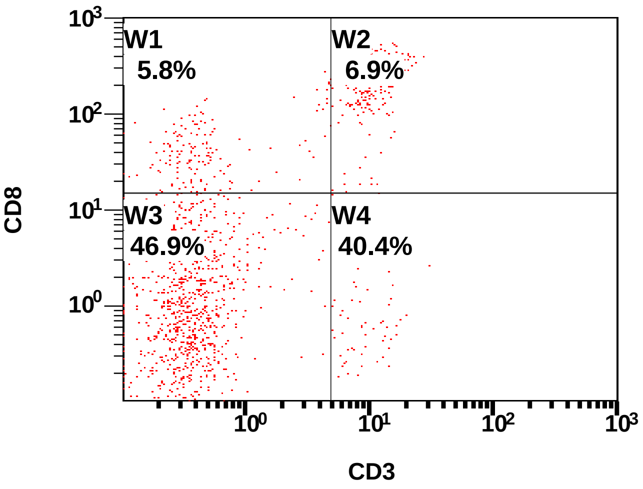

\] 5 day Hsp70 1d CD3 CD4 CD8 00012769 2020-01-29 6(F1)[Ungated] 5 day Hsp70 1d CD3 CD4 CD8 00012769 2020-01-29 613.LMD : SS Lin

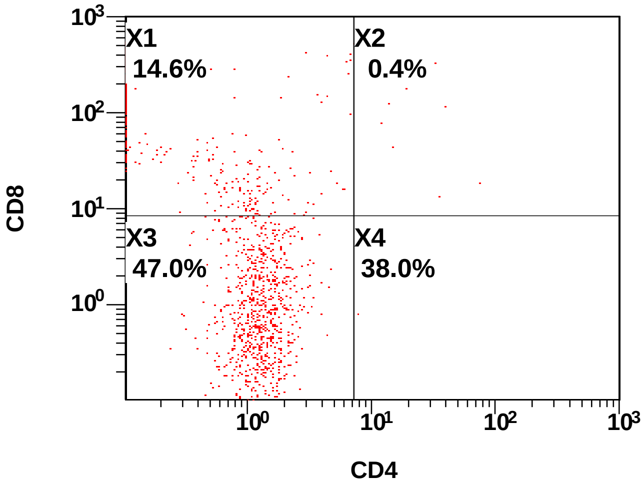

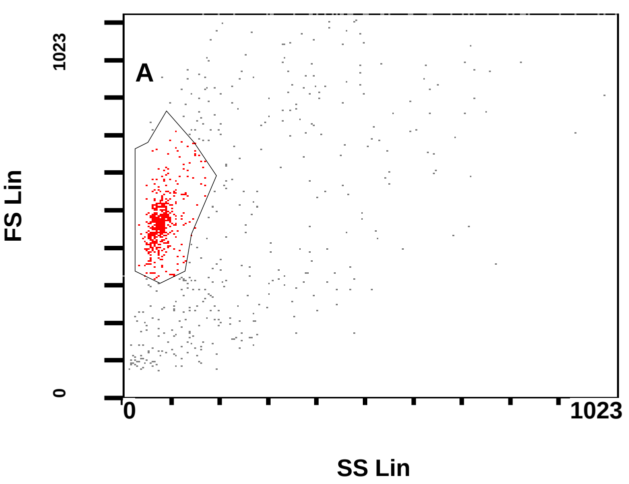

1)[A] 5 day Hsp70 1d CD3 CD4 CD8 00012769 2020-01-29 61:(F1)[A] 5 day Hsp70 1d CD3 CD4 CD8 00012769 2020-01-29 613.LMD : FL2 Log

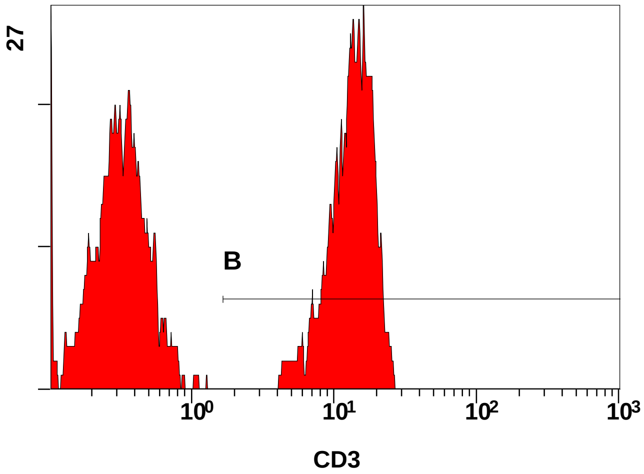

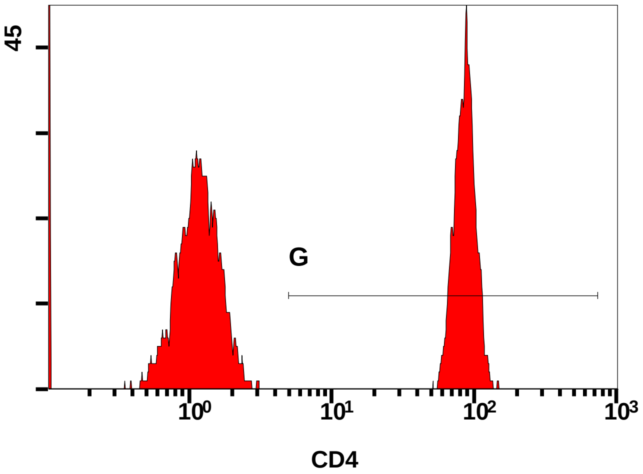

Institution: IBG
Protocol: 3P Tanya lymph 240120.PRO
Listmode Replay: New Protocol
Analysis Date: 20-Apr-2021, 20:04:59
Settings File: 3P Tanya lymph 240120.PRO, 29-Jan-2020, 16:01:52
Listmode File: 5 day Hsp70 1d CD3 CD4 CD8 00012769 2020-01-29 613.LMD

Run Date: 29-Jan-20, 16:02:09
Sample ID: 5 day Hsp70 1d
User ID: Yashin
Acquisition Time/Events: 12.6s / 10000 (PROTOCOL)

Instrument SN: AK02006 Software Version: CXP 2.2

:1)[A] 5 day Hsp70 1d CD3 CD4 CD8 00012769 2020-01-29 613.LMD : FL4 Log ·

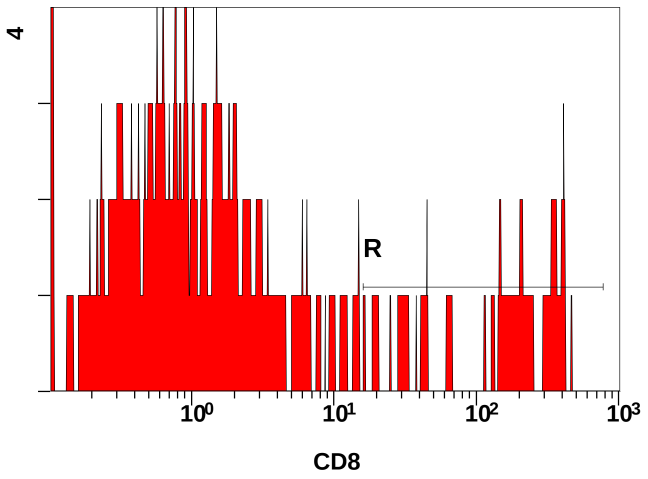

Statistical Analysis

PROGRAM INFORMATION

File:- 5 day Hsp70 1d CD3 CD4 CD8 00012769 2020-01-29 613.LMD					
Gate:- A [A]					
Compensation:- Advanced					
Filename:- 5 day Hsp70 1d CD3 CD4 CD8 00012769 2020-01-29 613.LMD					
Mean Calculation Method:-LOG-LOG					
Region	Number	%Total	%Gated	X-Mean	Y-Mean
ALL	5398	53.98	100.00	6.84	33.9
ALL	5398	53.98	100.00	33.9	###
ALL	5398	53.98	100.00	6.84	###
ALL	5398	53.98	100.00	6.84	20.9
ALL	5398	53.98	100.00	20.9	###
ALL	5398	53.98	100.00	33.9	20.9
B	2669	26.69	49.44	13.5	###
G	2083	20.83	38.59	86.1	###
R	676	6.76	12.52	161	###
U1	16	0.16	0.30	1	40.6
U2	2067	20.67	38.29	14.7	86.5
U3	2711	27.11	50.22	0.352	1.18
U4	604	6.04	11.19	9.31	0.657
W1	312	3.12	5.78	0.823	55.9
W2	375	3.75	6.95	11	244
W3	2532	25.32	46.91	0.447	1.45
W4	2179	21.79	40.37	14.4	0.175
X1	788	7.88	14.60	0.565	135
X2	19	0.19	0.35	22	213
X3	2539	25.39	47.04	1.27	0.975
X4	2052	20.52	38.01	87.2	0.111

File:- 5 day Hsp70 1d CD3 CD4 CD8 00012769 2020-01-29 613.LMD					
Gate:- Ungated					
Compensation:- Advanced					
Filename:- 5 day Hsp70 1d CD3 CD4 CD8 00012769 2020-01-29 613.LMD					
Mean Calculation Method:-LOG-LOG					
Region	Number	%Total	%Gated	X-Mean	Y-Mean
ALL	10000	100.00	100.00	195	512
A	5398	53.98	53.98	78.6	474
