# Supplementary material for: Hsp70 Interacts with the TREM-1 Receptor Expressed on Monocytes and Thereby Stimulates Generation of Cytotoxic Lymphocytes Active against MHC-Negative Tumor Cells
Source: Int J Mol Sci. 2021 Jun 26;22(13):6889. doi: 10.3390/ijms22136889 (PMC8267615; doi:10.3390/ijms22136889)
Supplement: Supplementary file 1 [file ijms-22-06889-s001.zip › Suppl5/Day 6 CD16CD56.PDF]

3P Tanya lymph 240120.PRO : CXP

Institution: IBG
Protocol: 3P Tanya lymph 240120.PRO
Listmode Replay: New Protocol
Analysis Date: 20-Apr-2021, 20:08:26
Settings File: 3P Tanya lymph 240120.PRO, 30-Jan-2020, 14:58:42
Listmode File: 6 day Hsp70 1 d CD16 CD56 00012774 2020-01-30 618.LMD

Run Date: 30-Jan-20, 14:58:59
Sample ID: 6 day Hsp70 1 d
User ID: Yashin
Acquisition Time/Events: 8.7s / 10000 (PROTOCOL)

Instrument SN: AK02006 Software Version: CXP 2.2

A] 6 day Hsp70 1 d CD16 CD56 00012774 2020-01-30 618(F1)[Ungated] 6 day Hsp70 1 d CD16 CD56 00012774 2020-01-30 618.LMD : SS Lin/

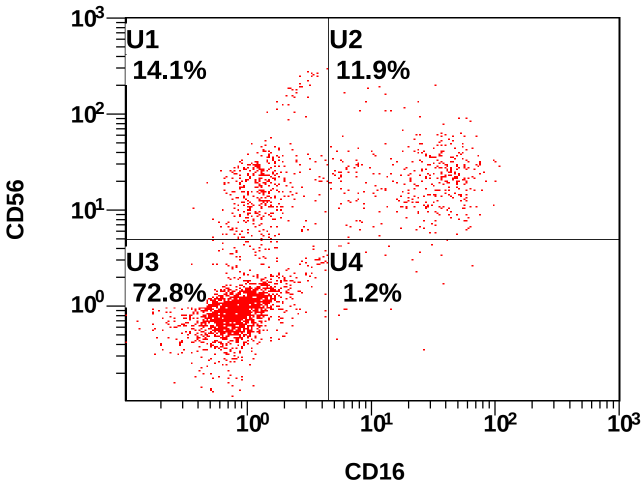

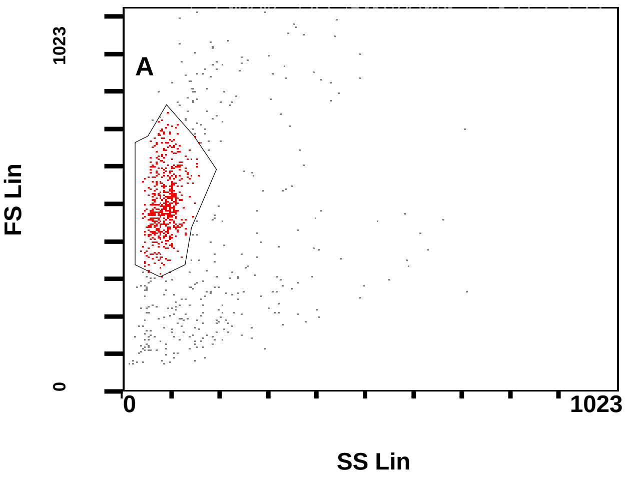

F1)[A] 6 day Hsp70 1 d CD16 CD56 00012774 2020-01-30 618.I(F1)[A] 6 day Hsp70 1 d CD16 CD56 00012774 2020-01-30 618.LMD : FL2 Log

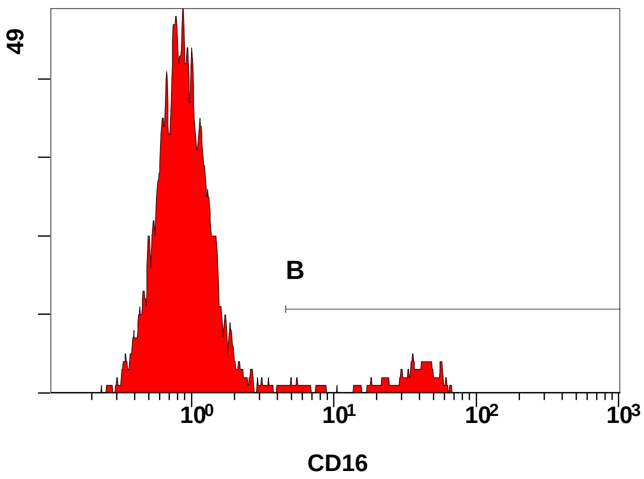

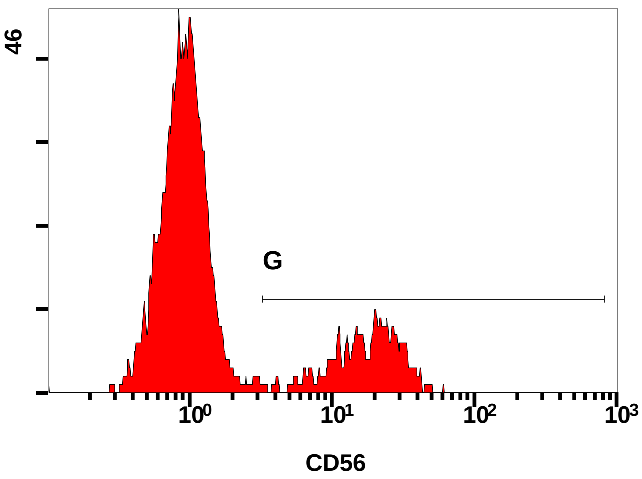

Statistical Analysis

PROGRAM INFORMATION

File:- 6 day Hsp70 1 d CD16 CD56 00012774 2020-01-30 618.LMD

Gate:- A [A]

Compensation:- Advanced

Filename:- 6 day Hsp70 1 d CD16 CD56 00012774 2020-01-30 618.LMD

Mean Calculation Method:-LOG-LOG

Region	Number	%Total	%Gated	X-Mean	Y-Mean
ALL	6323	63.23	100.00	4.91	8.09
ALL	6323	63.23	100.00	4.91	###
ALL	6323	63.23	100.00	8.09	###
B	828	8.28	13.10	31	###
G	1742	17.42	27.55	26.8	###
U1	889	8.89	14.06	1.48	27.4
U2	754	7.54	11.92	32.5	29.1
U3	4602	46.02	72.78	0.889	1.01
U4	78	0.78	1.23	14.8	2.79

File:- 6 day Hsp70 1 d CD16 CD56 00012774 2020-01-30 618.LMD

Gate:- Ungated

Compensation:- Advanced

Filename:- 6 day Hsp70 1 d CD16 CD56 00012774 2020-01-30 618.LMD

Mean Calculation Method:-LOG-LOG

Region	Number	%Total	%Gated	X-Mean	Y-Mean
ALL	10000	100.00	100.00	151	506
A	6323	63.23	63.23	85.6	506
