# Supplementary material for: Hsp70 Interacts with the TREM-1 Receptor Expressed on Monocytes and Thereby Stimulates Generation of Cytotoxic Lymphocytes Active against MHC-Negative Tumor Cells
Source: Int J Mol Sci. 2021 Jun 26;22(13):6889. doi: 10.3390/ijms22136889 (PMC8267615; doi:10.3390/ijms22136889)
Supplement: Supplementary file 1 [file ijms-22-06889-s001.zip › Suppl5/Day 6 CD3 -4-8.PDF]

3P Tanya lymph 240120.PRO : CXP

Institution: IBG
Protocol: 3P Tanya lymph 240120.PRO
Listmode Replay: New Protocol
Analysis Date: 20-Apr-2021, 20:05:19
Settings File: 3P Tanya lymph 240120.PRO, 30-Jan-2020, 15:00:21
Listmode File: 6 day Hsp70 1d CD3 CD4 CD8 00012775 2020-01-30 619.LMD

Run Date: 30-Jan-20, 15:00:38
Sample ID: 6 day Hsp70 1d
User ID: Yashin
Acquisition Time/Events: 12.2s / 10000 (PROTOCOL)

Instrument SN: AK02006 Software Version: CXP 2.2

\] 6 day Hsp70 1d CD3 CD4 CD8 00012775 2020-01-30 61(F1)[A] 6 day Hsp70 1d CD3 CD4 CD8 00012775 2020-01-30 619.LMD : FL1 Log/FL

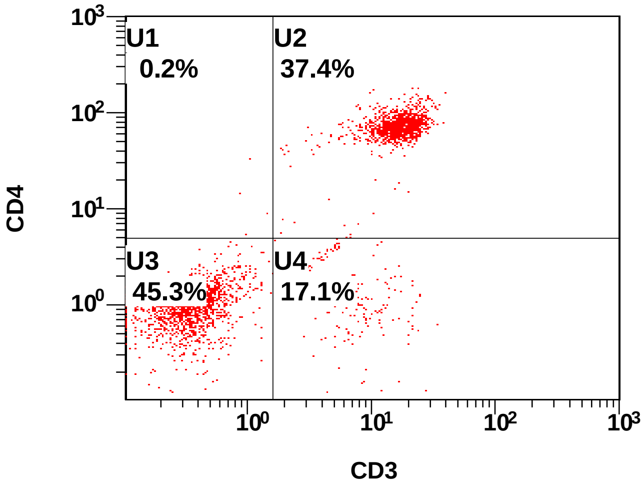

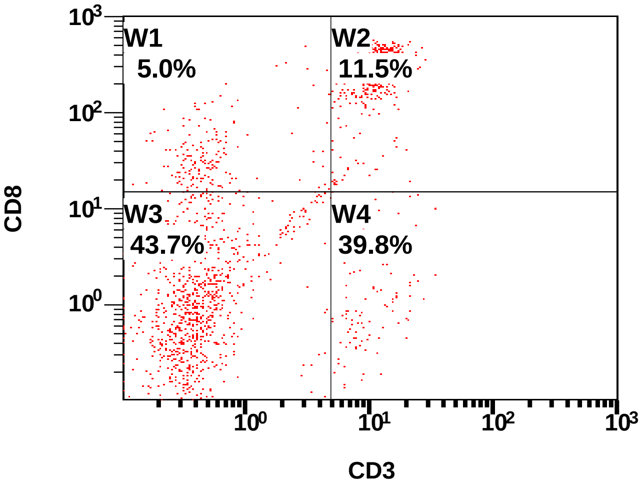

\] 6 day Hsp70 1d CD3 CD4 CD8 00012775 2020-01-30 6(F1)[Ungated] 6 day Hsp70 1d CD3 CD4 CD8 00012775 2020-01-30 619.LMD : SS Lin

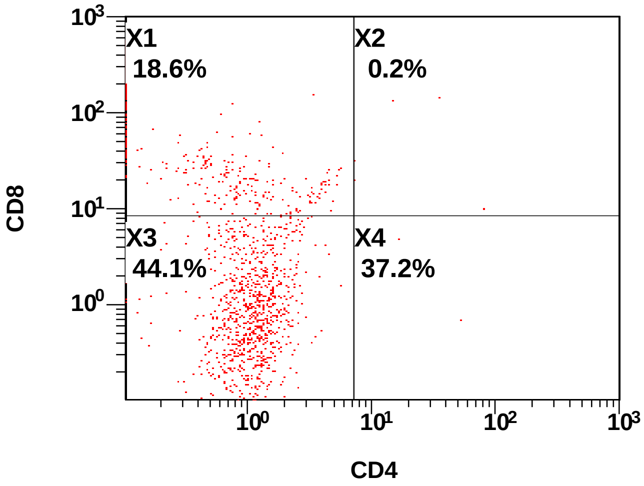

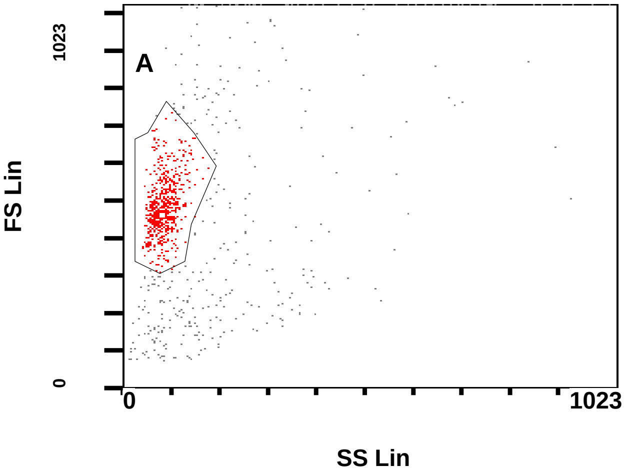

1)[A] 6 day Hsp70 1d CD3 CD4 CD8 00012775 2020-01-30 61(F1)[A] 6 day Hsp70 1d CD3 CD4 CD8 00012775 2020-01-30 619.LMD : FL2 Log

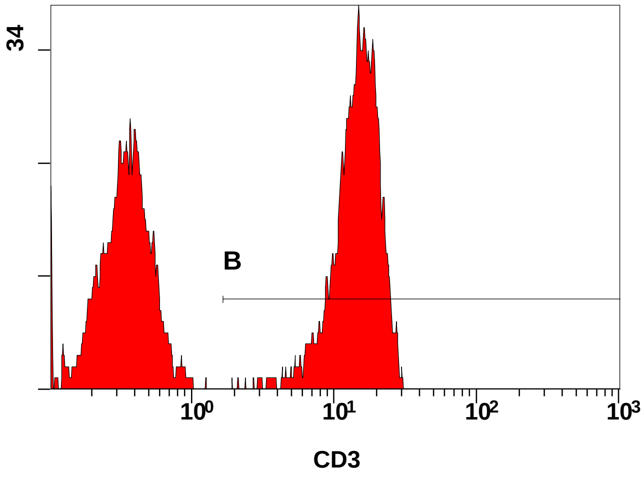

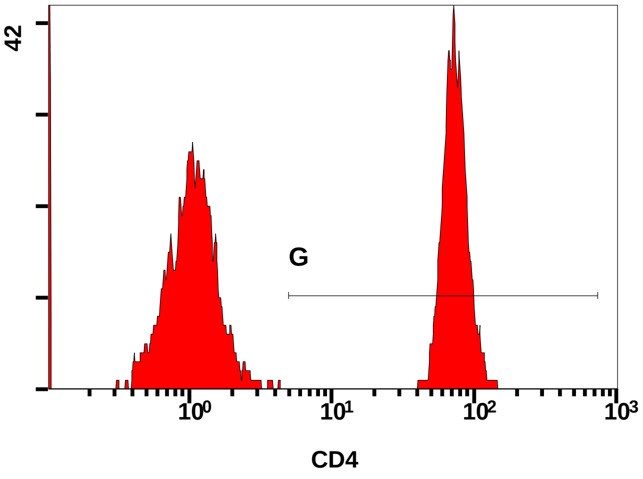

Institution: IBG
Protocol: 3P Tanya lymph 240120.PRO
Listmode Replay: New Protocol
Analysis Date: 20-Apr-2021, 20:05:19
Settings File: 3P Tanya lymph 240120.PRO, 30-Jan-2020, 15:00:21
Listmode File: 6 day Hsp70 1d CD3 CD4 CD8 00012775 2020-01-30 619.LMD

Run Date: 30-Jan-20, 15:00:38
Sample ID: 6 day Hsp70 1d
User ID: Yashin
Acquisition Time/Events: 12.2s / 10000 (PROTOCOL)

Instrument SN: AK02006 Software Version: CXP 2.2

:1)[A] 6 day Hsp70 1d CD3 CD4 CD8 00012775 2020-01-30 619.LMD : FL4 Log ·

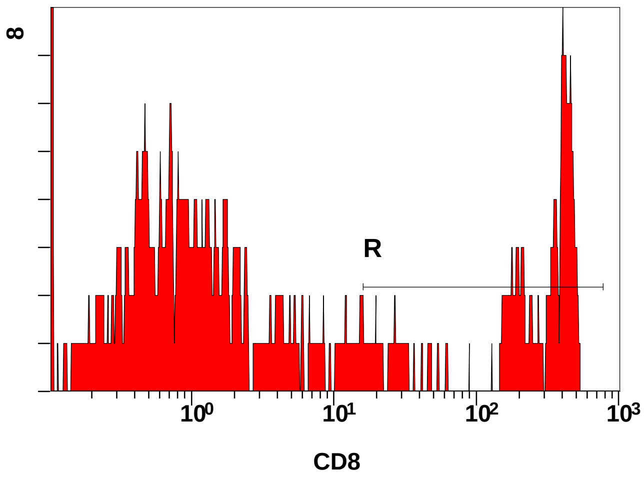

Statistical Analysis

PROGRAM INFORMATION

File:- 6 day Hsp70 1d CD3 CD4 CD8 00012775 2020-01-30 619.LMD					
Gate:- A [A]					
Compensation:- Advanced					
Filename:- 6 day Hsp70 1d CD3 CD4 CD8 00012775 2020-01-30 619.LMD					
Mean Calculation Method:-LOG-LOG					
Region	Number	%Total	%Gated	X-Mean	Y-Mean
ALL	6209	62.09	100.00	8.13	28.8
ALL	6209	62.09	100.00	28.8	###
ALL	6209	62.09	100.00	8.13	###
ALL	6209	62.09	100.00	8.13	37.6
ALL	6209	62.09	100.00	37.6	###
ALL	6209	62.09	100.00	28.8	37.6
B	3381	33.81	54.45	14.6	###
G	2336	23.36	37.62	75	###
R	1005	10.05	16.19	227	###
U1	13	0.13	0.21	1.16	23.2
U2	2324	23.24	37.43	16.5	75.2
U3	2812	28.12	45.29	0.383	1.06
U4	1060	10.60	17.07	10.5	0.642
W1	308	3.08	4.96	0.849	52.9
W2	716	7.16	11.53	11.9	296
W3	2716	27.16	43.74	0.539	1.51
W4	2469	24.69	39.76	16.3	0.266
X1	1157	11.57	18.63	0.576	198
X2	12	0.12	0.19	23.2	134
X3	2737	27.37	44.08	1.14	1.04
X4	2308	23.08	37.17	75.7	0.124

File:- 6 day Hsp70 1d CD3 CD4 CD8 00012775 2020-01-30 619.LMD					
Gate:- Ungated					
Compensation:- Advanced					
Filename:- 6 day Hsp70 1d CD3 CD4 CD8 00012775 2020-01-30 619.LMD					
Mean Calculation Method:-LOG-LOG					
Region	Number	%Total	%Gated	X-Mean	Y-Mean
ALL	10000	100.00	100.00	160	497
A	6209	62.09	62.09	84.8	493
